# Face and edge directed self-assembly of Pd_12_ tetrahedral nano-cages and their self-sorting†
†Electronic supplementary information (ESI) available: Experimental details, NMR, ESI-MS spectra and X-ray data in CIF format. CCDC 1471523, 1471434 and 1471478. For ESI and crystallographic data in CIF or other electronic format see DOI: 10.1039/c6sc02012g


**DOI:** 10.1039/c6sc02012g

**Published:** 2016-05-20

**Authors:** Prodip Howlader, Partha Sarathi Mukherjee

**Affiliations:** a Inorganic and Physical Chemistry Department , Indian Institute of Science , Bangalore-560012 , India . Email: psm@ipc.iisc.ernet.in ; Fax: +91-80-23601552 ; Tel: +91-80-22933352

## Abstract

Reactions of a *cis*-blocked Pd(ii) 90° acceptor [*cis*-(tmeda)Pd(NO_3_)_2_] (**M**) with 1,4-di(1*H*-tetrazol-5-yl)benzene (**H_2_L^1^**) and [1,3,5-tri(1*H*-tetrazol-5-yl)benzene] (**H_3_L^2^**) in 1 : 1 and 3 : 2 molar ratios respectively, yielded soft metallogels **G1** and **G2** [tmeda = *N*,*N*,*N*′,*N*′-tetramethylethane-1,2-diamine]. Post-metalation of the gels **G1** and **G2** with **M** yielded highly water-soluble edge and face directed self-assembled Pd_12_ tetrahedral nano-cages **T1** and **T2**, respectively. Such facile conversion of Pd(ii) gels to discrete tetrahedral metallocages is unprecedented. Moreover, distinct self-sorting of these two tetrahedral cages of similar sizes was observed in the self-assembly of **M** with a mixture of **H_2_L^1^** and **H_3_L^2^** in aqueous medium. The edge directed tetrahedral cage (**T1**) was successfully used to perform Michael reactions of a series of water insoluble nitro-olefins assisted by encapsulation into the cage in aqueous medium.

## Abstract 2

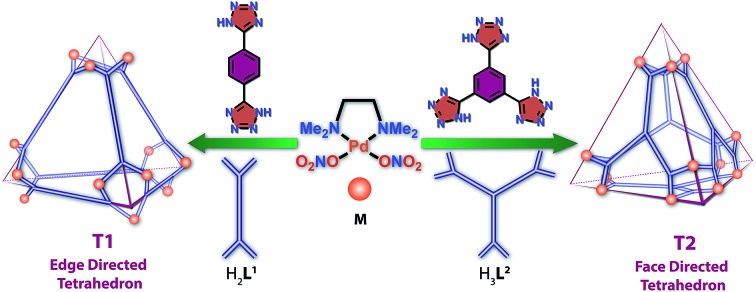
A di-tetrazole ligand was used to occupy the edges of a tetrahedron to generate an edge-directed self-assembled Pd_12_ water soluble cage which was used as a vessel to encapsulate aromatic nitro-olefins. A face directed Pd_12_ tetrahedral cage was also constructed occupying the triangular faces of the tetrahedron by a tri-tetrazole ligand.

## Introduction

Nature, especially in biological systems, has an extraordinary ability to develop complex and functional molecular assemblies employing reversible non-covalent interactions.^1^ These natural aesthetic examples have enticed synthetic chemists over the past several years to develop potential synthetic protocols to produce myriad complex assemblies employing non-covalent interactions like H-bonding, π–π interactions and stronger metal-ligand coordination.^2^–^4^ In principle, by tuning these directional non-covalent driving forces one can construct supramolecular polymers as well as discrete molecular assemblies. Such ordered and self-organized polymers may go through gelation in the presence of strong inter-molecular interactions (network formation).^5^ The three-dimensional fibrous network structure generated by the self-assembly of the gelator molecules may impart solid-like properties in the gels, which make them worthy candidates for substrate recognition, catalysis and biomedical applications.^6^ Meanwhile, discrete molecular architectures are also potential candidates for stabilizing reactive intermediates, sensing, and host-guest chemistry, as well as cavity-induced ‘organic transformations’.^7^

Introducing multiple interactions in a single system and tuning them by varying different parameters such as solvent type, temperature and stoichiometry to construct desired molecular architectures (discrete or polymeric), including their transformation, is interesting and provides diverse functional materials. Discrete supramolecular metallocages have been designed, mainly employing pyridyl, imidazole and carboxylate linkers.^8^ Polytetrazoles are not preferred linkers in designing molecular cages due to difficulty in predicting the final structure because of the multiple nitrogen atoms. The N–H moiety in the tetrazole ring promotes many metal complexes of polytetrazoles to form metallogels by intermolecular H-bonding.^9^

The coordination-driven self-assembly strategy has inspired chemists in the past two decades to design basic 3D structures of high symmetry and well defined shapes and sizes.7b,^10^ A tetrahedron is one of the common 3D geometries. Several tetrahedral molecular cages have been reported using mainly octahedral metal ions with a few examples of analogous cages of lanthanides with a higher coordination number, though their solubility is restricted to organic solvents in the majority of cases.^11^,^12^ Due to geometric restriction, square planar metal ions like Pd(ii) have not been explored much for the design of regular tetrahedral cages.

**Scheme 1:**
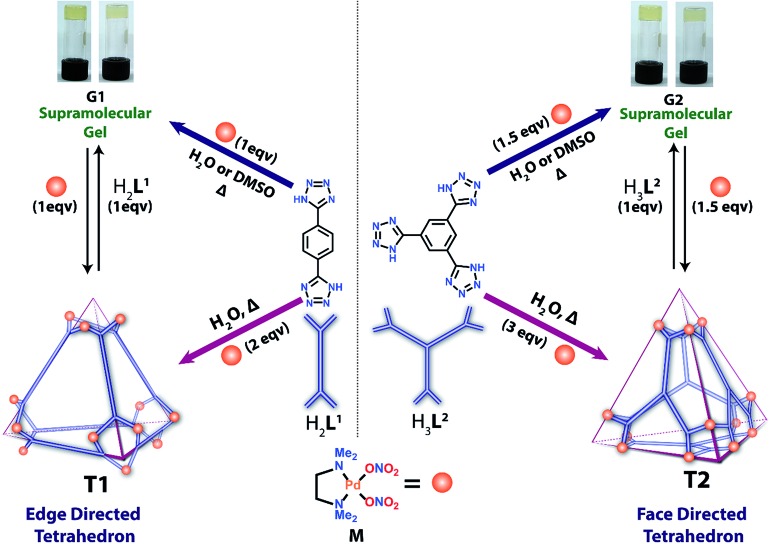
Schematic representation of the synthesis of metallogels and their facile conversion to 3D tetrahedral nano-cages.

Herein, we report the formation of a supramolecular Pd(ii) metallogel (**G1**) upon 1 : 1 treatment of **H_2_L^1^** [1,4-di(1*H*-tetrazol-5-yl)benzene] with *cis*-(tmeda)Pd(NO_3_)_2_ (**M**) in water or DMSO (Scheme 1). Post-metalation of **G1** with **M** in water/or DMSO caused deprotonation of the N–H moieties and transformed the gel into a highly water-soluble unusual tetrahedral **M_12_L^1^_6_** (**T1**) cage where the donors (**L^1^**) occupy the six edges of the tetrahedron. Such post-metalation was expected to form an open 2D square **M_8_L^1^_4_** as observed using pyrazole linkers instead of a closed 3D tetrahedral cage.^13^ This unusual outcome enticed our attention towards a face directed tetrahedron to examine the generality of designing tetrahedral cages of square planar metal ions employing polytetrazole donors. Replacement of **H_2_L^1^** with **H_3_L^2^** [1,3,5-tri(1*H*-tetrazol-5-yl)benzene] in the above-mentioned two-step reaction afforded a Pd(ii) gel (**G_2_**) followed by a water soluble chiral tetrahedral cage **M_12_L^2^_4_** (**T_2_**) (Scheme 1), where the four triangular faces of the tetrahedron were occupied by **L^2^**. **T1** and **T2** are unusual from a symmetry point of view.11b,^14^ The spatial orientations of the linkers **L^1^** and **L^2^** enable the final assemblies (**T1** and **T2**) to adopt a specific geometry with a loss of symmetry to become chiral cages of square planar Pd(ii) using achiral building blocks.

## Results and discussion

### Synthesis and characterization of metallogels

**Fig. 1:**
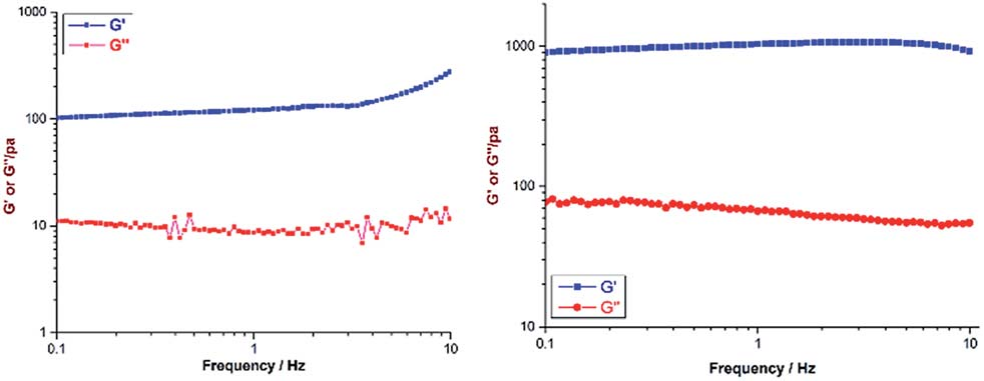
*G*′′ and *G*′ *vs.* frequency sweep for **G1** (left) and **G2** (right).

Both the ligands (**H_2_L^1^** and **H_3_L^2^**) were prepared from their cyano derivatives following the reported procedure.^9^ Supramolecular hydrogel **G1** was prepared by adding an aqueous yellow solution of the acceptor **M** to **H_2_L^1^** in a 1 : 1 molar ratio with a total weight percentage of 2. The mixture was heated with stirring at 60 °C for 2 h to give a transparent solution, and subsequent cooling to room temperature yielded the gel **G1** (Scheme 1). A similar gel was also obtained when DMSO was used as a solvent in the above reaction. The viscoelastic nature of the gel was characterized by two types of dynamic rheology^15^ experiments: (a) frequency sweep at a constant stress of 1.0 Pa and (b) stress sweep at a constant frequency of 1.0 Hz. The stiffness of the gel (*G*′/*G*′′) can be measured from the first experiment whereas fragility (yield stress) can be measured from the second one. For **G1** and **G2** these experimental values are quite small (ESI†), which suggests the formation of a weak gel (Fig. 1). Finally, field emission scanning electron microscopy of the xerogels revealed that the formation of a fibrous nanostructure (tertiary structure) is responsible for gelation (ESI†).

### Synthesis and characterization of nano-cages

Treatment of the metallogel **G1** with an aqueous solution of one molar equivalent of **M** at 60 °C for 3 h yielded **T1**. To investigate the extent of the deprotonation of the ligand **H_2_L^1^** during the self-assembly process, a pH monitored self-assembly reaction was performed in water with 2 molar equivalents of **M** and one molar equivalent of **H_2_L^1^**. The change in pH of the reaction mixture after the consumption of all the ligand **H_2_L^1^** (ESI†) clearly suggests deprotonation of both of the N–H protons of **H_2_L^1^**. Evaporation of the solvent under vacuum and subsequent washing of the resulting solid with acetone yielded the final product in pure form. In the case of the DMSO gel, a similar procedure was followed with a DMSO solution of **M**, and finally, **T1** was obtained as a white solid upon treating the solution with ethyl acetate. ^1^H NMR analysis of the product (**T1**) in D_2_O displayed a single peak at 9.1 ppm, which is significantly downfield shifted compared to the peak for free **H_2_L^1^** (ESI†). ESI-MS analysis in water indicated a [12 + 6] composition of **M** and **L^1^** in the final product (**T1**) by the appearance of two major peaks at *m*/*z* = 1110 and 1500 with isotropic distribution patterns corresponding to the fragments [**T1**(NO_3_)_8_]^4+^ and [**T1**(NO_3_)_9_]^3+^, respectively (ESI†).

**T2** was synthesized following the above-mentioned procedure using **H_3_L^2^** instead of **H_2_L^1^**. The metallogel **G2** was obtained by treating **H_3_L^2^** and **M** in a 2 : 3 molar ratio in DMSO or water. **G2** was converted to **T2** by post-metalation treatment with **M**. The presence of a singlet at 10.05 ppm in ^1^H NMR indicated the formation of a single and symmetrical product (ESI†). ESI-MS analysis displayed two major peaks at *m*/*z* = 1070.64 and *m*/*z* = 1048.53 with the isotropic distribution patterns corresponding to the fragments [**T2**(NO_3_)_8_]^4+^ and [**T2**(NO_3_)_9_]^3+^, which confirmed the [12 + 4] composition of **M** and **L^2^** in the final assembly **T2** (ESI†).

**Fig. 2:**
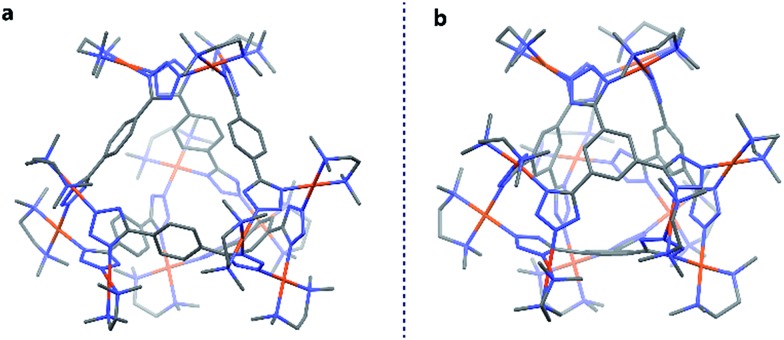
Crystal structures of the tetrahedra **T1** and **T2** (all the hydrogen atoms are omitted for clarity). Colour codes: grey: carbon, blue: nitrogen and brown: palladium.

**Fig. 3:**
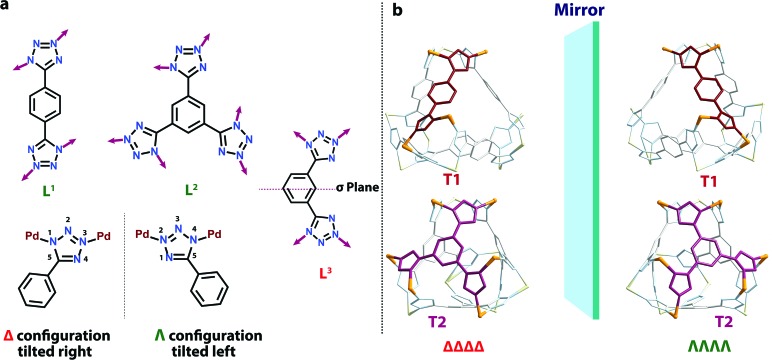
(a) Coordination modes of **L^1^L^2^** and **L^3^** in the final assemblies. (b) Representations of the enantiomeric forms of **T1** (top) and **T2** (bottom).

**Fig. 4:**
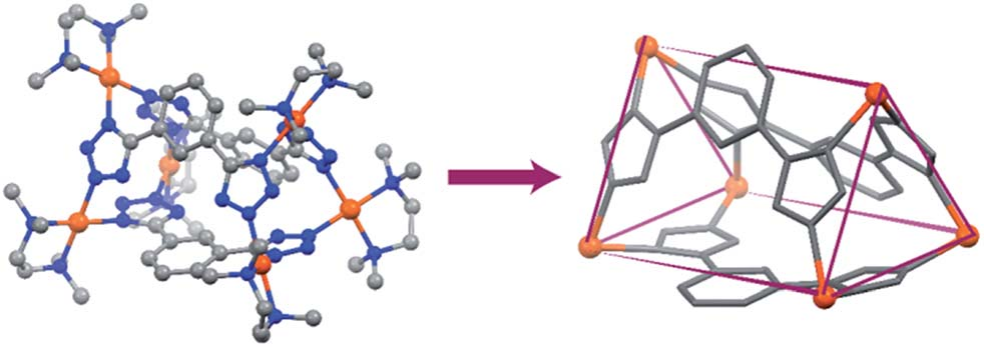
Crystal structure of the achiral molecular prism **P**. Colour codes: grey, carbon; blue, nitrogen; and brown, palladium.

Finally, both the cages were successfully crystallized by diffusion of acetone vapour into the aqueous solutions of the cages. Single crystal XRD analysis of both **T1** and **T2** unequivocally confirmed the formation of 3D tetrahedral cages (Fig. 2). **T1** was crystallized in the *C*2/*c* space group and each vertex corner of the tetrahedron contains 3 Pd(ii) acceptors connected by the nitrogens of the tetrazole moieties of the linkers to form a Pd_3_ triangle and the ligands **L^1^** are fastened by two corners along the edges. Whereas **T2** was crystallized in the *I*222 space group and each vertex corner of the tetrahedron contains three acceptors and the linker **L^2^** is tied by three corners along the face. So, in the case of **T1** the ligands occupy the edges of the tetrahedron while in **T2** the donors occupy the triangular faces of the tetrahedron. **L^1^** and **L^2^** bind the Pd(ii) centers through the nitrogens of the 1 and 3 positions of the tetrazole rings (Fig. 3). Such binding modes of the tetrazole ligands (for **L^1^** and **L^2^**) make them special in terms of “symmetry breaking”,11*b* because **L^1^** and **L^2^** have no plane of symmetry when they are coordinated to metal centers, except in the molecular plane, which no longer exists in the 3D cage. Now if we consider the 1,3 binding mode, the tetrazole moiety is residing on the right with respect to the phenyl ring (Fig. 3). If we consider this geometry as a Δ configuration; the 2,5 binding mode brings the tetrazole moiety over to the left side, which may be represented as a Λ configuration. Crystal structures of **T1** and **T2** (CCDC ; 1471523 and ; 1471434 †) clearly displayed that, in a particular nano-cage, all the linkers have a similar type of coordination mode with the Pd(ii) acceptors as shown in Fig. 4. Hence, the handedness of all the Pd_3_ triangles (vertices of each tetrahedron) is the same, either ΔΔΔΔ or ΛΛΛΛ. Such spatial arrangements of the donors and acceptors make the resulting cages chiral even without using any chiral building blocks. As the cage **T1** was crystallized in a centrosymmetric space group (*C*2/*c*), the crystal packing exposed the presence of both enantiomers along the *c* glide plane in the unit cell.

Although **T2** was crystallized in a chiral (*I*222) space group, the Flack parameter was evaluated to be 0.5, which is due to the formation of an inversion twin *i.e.* a racemic mixture. Nonetheless, linker **L^3^** forms a prismatic structure **M_6_L^3^_3_** (**P**) (CCDC ; 1471478 †).This was synthesized in a similar way as followed for **T1** and **T2**. The binding mode of **L^3^** is shown in Fig. 3. It forms two Pd_3_ triangles with opposing handedness yielding an achiral geometry (Fig. 4). So, the achiral or chiral nature of the final assemblies is controlled by the handedness of the Pd_3_ triangular units.

### Variable-temperature ^1^H NMR study of **T1**

**Fig. 5:**
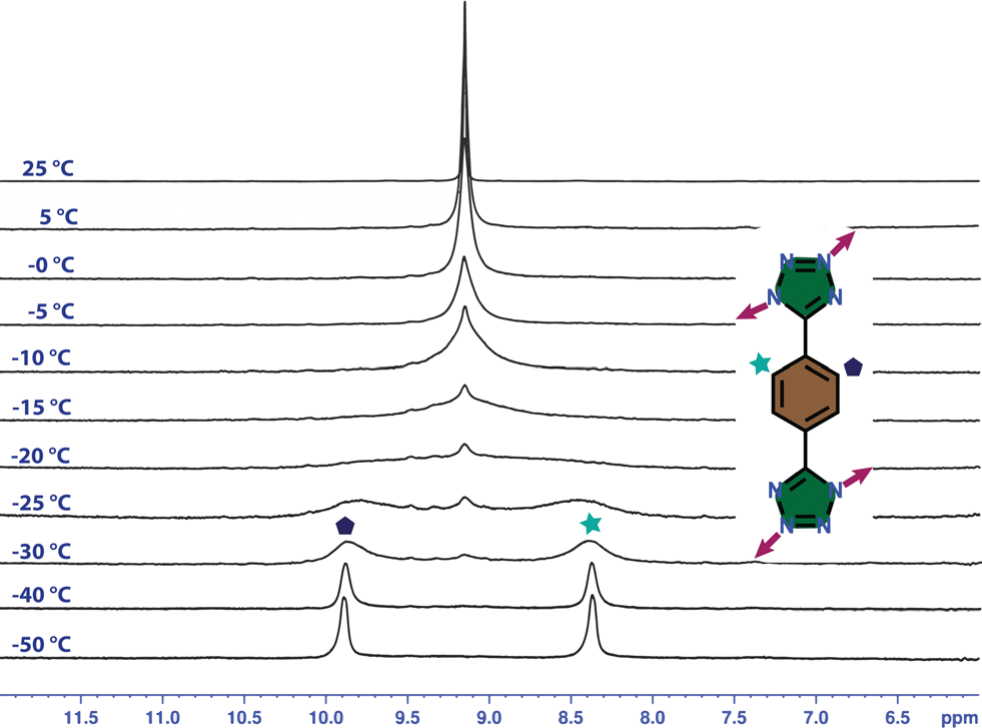
Temperature dependent ^1^H NMR spectra of **T1** in CD_3_OD showing the presence of two different sets of phenyl protons at low temperature.

Interestingly, the ^1^H NMR study of **T1** at room temperature does not support the asymmetric nature of the solid-state XRD structure. The single peak at 9.1 ppm is due to the possibility of rotation of the phenyl ring with a frequency higher than the frequency of the NMR technique at room temperature. Variable temperature NMR spectra at low temperature were needed to confirm this phenomenon. Although the cage was synthesized in water or DMSO, it is soluble in MeOH. This allowed us to study ^1^H NMR of the cage at –50 °C (Fig. 5), which displayed two different peaks at 8.5 and 9.8 ppm. Moreover, a DOSY NMR study at that temperature showed the presence of a single product. Upon increasing the temperature, the peaks started to merge together and at around –20 °C these peaks disappeared and the original peak at 9.1 ppm appeared.

### Self-sorting experiment of **T1** and **T2**

**Fig. 6:**
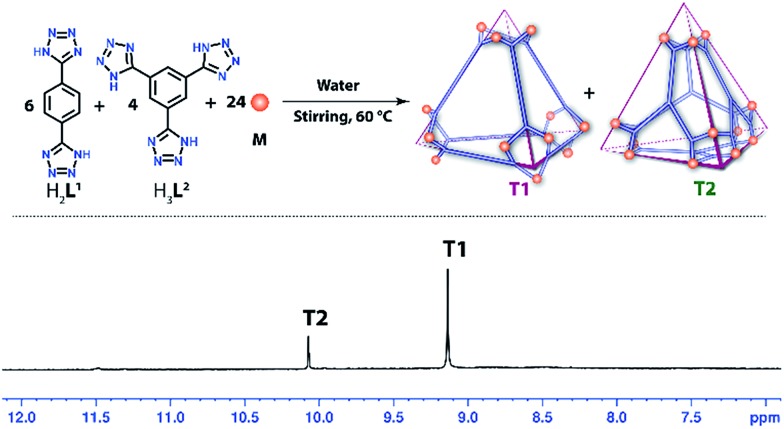
Self-sorting of **T1** and **T2** from a mixture of **M**, **H_2_L^1^** and **H_3_L^2^** in water (top) and the ^1^H NMR spectrum of the resulting mixture.

As both the linkers **L^1^** and **L^2^** form tetrahedral cages of almost equivalent shape and size with complementary building units (edge directed and face directed respectively), we were curious to know whether the presence of both the donors (**L^1^** and **L^2^**) in a single reaction mixture would result in these two individual tetrahedral cages by a self-sorting process or a complicated multicomponent product. To investigate this, a self-assembly experiment was carried out in water taking **M**, **H_2_L^1^** and **H_3_L^2^** in a 24 : 6 : 4 molar ratio. The final product was isolated and characterized by ^1^H NMR and mass spectroscopy. The presence of two sharp singlets at 10.07 and 9.13 ppm in the ^1^H NMR spectra clearly indicates the formation of **T2** and **T1** in pure form without the presence of any byproduct (Fig. 6).

### Catalytic Michael addition reaction

The donors (**L^1^**) in the edge directed cage **T1** occupy the edges of the tetrahedron by joining two Pd_3_ triangles, keeping four triangular faces of the tetrahedron open, having dimensions of 6.8 Å × 6.9 Å, which may allow one or more aromatic guests to enter. Cage **T2** has complementary geometry, where all four faces of the tetrahedron are occupied by the linker **L^2^** leaving no open windows for aromatic guest encapsulation. The edges of this cage are also blocked by the methyl groups of the acceptor leaving no pathway for the entry of guests.

**Fig. 7:**
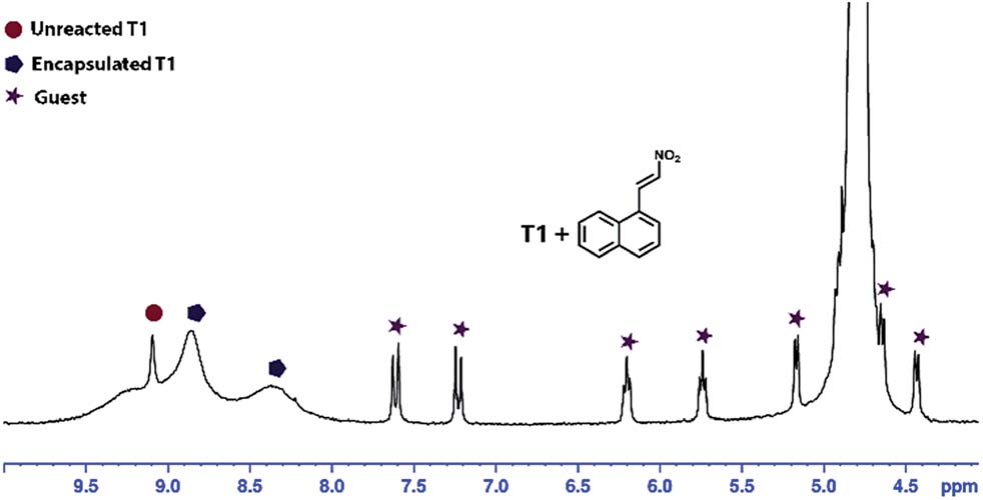
^1^H NMR spectrum of guest (**1**) encapsulated in **T1** (1 ⊂ **T1**) in D_2_O.

This structural nature of the cages motivated us to study the possibility of encapsulating a water insoluble aromatic guest. To verify our observation the aqueous solutions of the cages were treated with 1-(2-nitrovinyl)naphthalene (**1**). As anticipated, the aromatic guest (**1**) was encapsulated in **T1** which was identified by a change in the colour of the cage solution (colourless to light yellow) and was finally confirmed by ^1^H NMR spectroscopy (Fig. 7) where significant up-field shifts of the guest peaks (**1**) were noticed. The stoichiometry of cage *vs.* guest was evaluated to be 1 : 2 from the ^1^H NMR spectra (ESI†). The broad ^1^H NMR peaks for the encapsulated cage can be explained in terms of the rotational movement of the phenyl ring of the cage **T1**, which is restricted by the guest molecules present inside the cage. This host-guest binding was further confirmed by ^1^H DOSY NMR (ESI†), which showed identical diffusion coefficients for the guest (**1**) and the host cage.

**Fig. 8:**
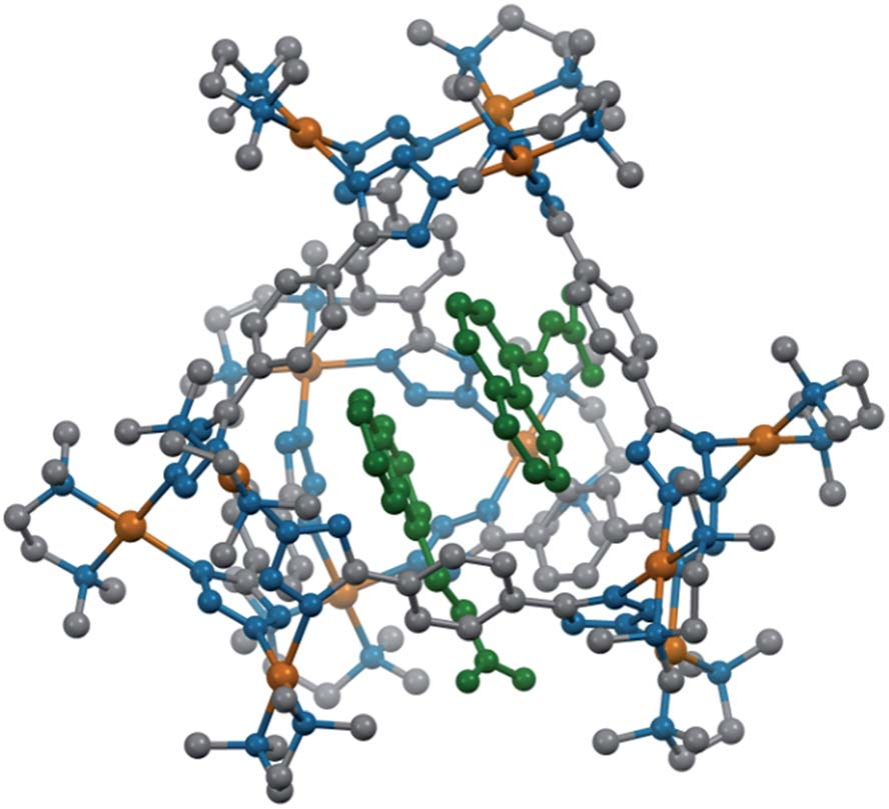
PM6 energy optimized structure of the guest encapsulated cage 1 ⊂ **T1**.

Several attempts to crystallize the guest encapsulated cage 1 ⊂ **T1** have so far been unsuccessful. The host-guest (1 : 2) complex was modeled and the structure was optimized by a semi-empirical method with a PM6 basis set. As the nitro group is more polar than the naphthyl group, it may lean outside the cavity. However, the naphthyl moieties are stabilized inside the hydrophobic cavity of the cage by π–π stacking interactions (Fig. 8).

This successful encapsulation of the aromatic nitro-alkene enabled it to be soluble in an aqueous medium and it was possible to carry out further organic transformation utilizing the cage **T1** in a phase transfer catalytic manner. Several reactions were studied using 1,3-dimethylbarbituric acid (**3**) and different nitro-alkenes (**2**) (Table 1). Although similar reactions were studied by us using a urea decorated functional self-assembled molecular prism in a heterogeneous manner,7*i* the present study was done in a homogeneous manner. The initial results presented in Table 1 indicate an enhancement of the yield in the presence of the cage **T1**. Cage **T2** didn't show any such encapsulation of nitro-olefins due to the absence of large open windows. Control reactions were carried out with **T2**, which indicated almost no catalytic effect on these reactions.

**Table 1:** Catalytic Michael addition reactions of 1,3-dimethylbabrbituric acid with different nitro-alkenes^*a*^

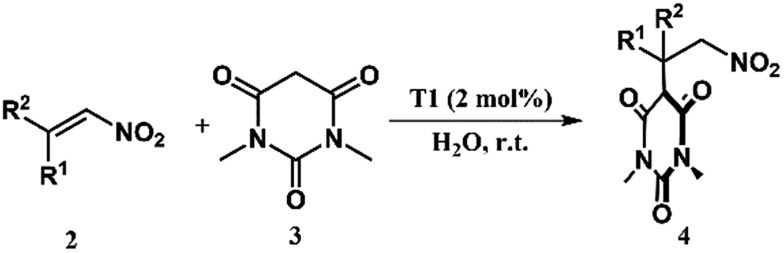							
Entry	R^1^	R^2^	Product	Time (min)	Yield^*b*^ (%)		
					Blank	With **T2**	With **T1**
1	1-Pyrenyl	H	4a	72 h	14	—	20
2	1-Naphthyl	H	4b	90	15	22	41
3	Ph	Me	4c	60	18	27	59
4	4-Me-Ph	H	4d	15	10	—	50
5	4-MeO-Ph	H	4e	30	13	20	54
6	2-Furanyl	H	4f	10	12	—	60

^*a*^Nitro-olefins **2** (0.02 mmol), 1,3-dimethylbarbituric acid **3** (0.02 mmol), catalyst **T1** (2 mol%), and water (1 mL), at r.t. with stirring.

^*b*^Crude yields were determined by ^1^H NMR studies.

## Conclusions

In conclusion, self-assembly of a Pd(ii) acceptor **M** with polytetrazole donors **H_2_L^1^** and **H_3_L^2^** in 1 : 1 and 3 : 2 molar ratios yielded supramolecular metallogels **G1** and **G2**, respectively. Post-metalation reactions of the gels with **M** transformed them to edge- and face-directed self-assembled water-soluble tetrahedral cages **T1** and **T2**. Reversible transformation of the cages to their corresponding parent gels was also achieved upon treating them with respective tetrazoles. To the best of our knowledge, such facile and reversible transformation of metallogels to discrete metallocages is unusual. Although pyridine, imidazole and carboxylates have been widely employed to obtain metallosupramolecular discrete architectures, **T1–T2** represent a unique class of tetrahedral architectures of square planar metal ions employing polytetrazoles as linkers. Interestingly, the coordination modes of the tetrazole units promote ‘symmetry breaking’ in these assemblies. Single crystal structures showed that the handedness of the Pd_3_ vertices formed by the tetrazole units is responsible for the ‘symmetry breaking’ which led to the formation of chiral nano-cages having *T* symmetry. However, the binding mode of **L^3^** imparts a mirror symmetry leading to an achiral prismatic structure **P**. The mixed-ligand self-assembly with **M** produced self-sorted cages **T1** and **T2** without any by-product. This clean self-sorting between the iso-structural tetrahedral cages of similar sizes in a complex mixture of **H_2_L^1^**, **H_3_L^2^** and **M** is reminiscent of the selectivity observed in nature. The open triangular windows in the edge directed tetrahedron (**T1**) enabled it to encapsulate water insoluble aromatic nitro-olefins in aqueous medium followed by Michael reactions of the nitro-olefins with 1,3-dimethybarbituric acid using phase transfer-type catalysis. The freshly prepared water-soluble tetrahedral cages provide a platform for the development of a new generation of functional molecular nanovessels employing polytetrazole donors for chemical reactions and encapsulation of various guests.

## Supplementary Material

Supplementary information

Crystal structure data
