# Correction: Face and edge directed self-assembly of Pd_12_ tetrahedral nano-cages and their self-sorting

**DOI:** 10.1039/c7sc90060k

**Published:** 2017-08-24

**Authors:** Prodip Howlader, Partha Sarathi Mukherjee

**Affiliations:** a Inorganic and Physical Chemistry Department , Indian Institute of Science , Bangalore-560012 , India . Email: psm@ipc.iisc.ernet.in ; Fax: +91-80-23601552 ; Tel: +91-80-22933352

## Abstract

Correction for ‘Face and edge directed self-assembly of Pd_12_ tetrahedral nano-cages and their self-sorting’ by Prodip Howlader *et al.*, *Chem. Sci.*, 2016, **7**, 5893–5899.



## 

An incorrect grant number was quoted for this paper and the corrected Acknowledgement section is as shown below:

P. S. M. thanks the DST (New Delhi) for the research grant [DST/SJF/CSA-01/2011-2012(G)]. P. H. is grateful to Mr Sayantan Chatterjee for the rheology experiments and also thankful to Mr Shaunak Chakraborty for fruitful discussion on the single crystal structure analysis.

The Royal Society of Chemistry apologises for these errors and any consequent inconvenience to authors and readers.

