# Supplementary material for: Face and edge directed self-assembly of Pd12 tetrahedral nano-cages and their self-sorting
Source: Chem Sci. 2016 May 20;7(9):5893–9. doi: 10.1039/c6sc02012g (PMC6024303; doi:10.1039/c6sc02012g)
Supplement: Supplementary file 1 [file SC-007-C6SC02012G-s001.pdf]

Electronic Supplementary Material (ESI) for Chemical Science.
This journal is © The Royal Society of Chemistry 2016

Supporting Information

Face and edge directed self-assembly of Pd_{12} tetrahedral nano-cages and their self-sorting

Prodip Howlader and Partha Sarathi Mukherjee*

Department of Inorganic and Physical Chemistry, Indian Institute of Science, Bangalore, 560012, Email: psm@ipc.iisc.ernet.in

Table of Content

Materials and methods…………………………………………………………..

Synthesis of the ligands **L^1**, **L^2** and **L^3**…………………………………………

Synthesis of **G1**, **G2** and their conversion to **T1** and **T2**……………………..

Synthesis of **P**…………………………………………………………………

Self-sorting experiment………………………………………………………….

Single crystal XRD experiments………………………………………………..

^{1}H NMR spectra of the ligands **L^1**, **L^2** and **L^3**………………………………….

Rheology experimental plots……………………………………………………

Scanning electron microscopic images…………………………………….

^{1}H NMR and ^{1}H DOSY NMR spectra of **T1** and **T2**………………………..

ESI-MS spectra of **T1** and **T2**………………………………………………….

^{1}H NMR and ^{1}H DOSY NMR spectra of **P**…………………………………….

Low-temperature NMR (^{1}H DOSY and COSY) of **T1**………………………..

ESI-MS spectra of the self-sorting reaction……………………………….

^{1}H, DOSY and COSY NMR spectra of **1⊂T1**……………………………….

^{1}H NMR data of the catalysis product……………………………………….

Materials and methods. All the reagents were purchased from different commercial sources and used without further purification. NMR spectra were recorded on a Bruker 400 MHz spectrometer and the chemical shifts (δ) in the ^1H NMR spectra are reported in ppm relative to tetramethylsilane (Me_4Si) as an internal standard (0.0 ppm) or proton resonance resulting from incomplete deuteration of the solvents $(CD_3)_2SO$ (2.51 ppm) and D_2O (4.79 ppm). Electrospray ionization mass spectrometry (ESI-MS) experiments were carried out on a ESI-MS spectra were recorded on a Q-TOF electrospray instrument. Dynamic rheological measurements were carried out with the gels and an AR 1000 rheometer (TA instruments) with a plate-plate (the rotor was serrated) geometry (20 mm diameter,400 μm gap). Scanning electron micrographs were recorded on an SERION instrument. Prior to the measurements a thin layer of the gels was taken on a silicon wafer and dried under vacuum and finally coated with a thin gold layer.

Synthesis of L^1, L^2 and L^3:

All the ligands L^1, L^2 and L^3 were synthesized following the reported procedure[1]. In a clean and dried round bottom flask 1,4-dicyanobenzene (10.0 mmol, 1.28 g), NaN_3 (60.0 mmol, 3.90 g) and trimethylamine hydrochloride (60.0 mmol, 8.25 g) were taken, to which 100 mL of toluene and 30 mL of methanol were added and the mixture was refluxed for 4 days. The resulting precipitate was filtered and dissolved in aqueous NaOH (1M). The clear colourless solution was then titrated with 1M HCl until it reached at pH ~4. The precipitated product was washed with water and methanol followed by drying under vacuum. Isolated yield 1.70 g (79%). ^1H NMR(DMSO-d_6) δ (ppm): 8.22 (s, 4H). L^2 and L^3 were also synthesized following the above-mentioned procedure. L^2: Isolated yield 84%. ^1H NMR(DMSO-d_6) δ (ppm): 8.82 (s, 3H). L^3: Isolated yield 80%. ^1H NMR(DMSO-d_6) δ (ppm): 8.79 (t, 1H), 8.24 (q, 2H), 7.85 (t, 1H).

Synthesis of G1:

In a cleaned and dried glass vial *cis*-(tmeda)Pd$(NO_3)_2$ (**M**) (0.04 mmol, 13.8 mg) and [1,4-di(1*H*-tetrazol-5-yl)benzene (**L^1**) (0.04 mmol, 8.6 mg) were taken followed by the addition of 1 mL water. The mixture was heated at 50 °C with stirring until all the solids dissolved. The solution was then heated at 50 °C for 6 h to form the hydrogel. Subsequently, the DMSO gel was synthesized by taking the exact amount of the reagents in 1 mL DMSO and heating it at 60 °C for 5 h.

Synthesis of G2:

In a dried glass vial **M** (0.04 mmol, 13.8 mg), 1,3,5-tri(1*H*-tetrazol-5-yl)benzene (**L^2**) (0.026 mmol, 7.5 mg) and 1 mL of water were taken. The heated at 50 °C with continuous stirring until L^2 consumed completely. The solution was then heated at 50 °C for 8 h to produce the hydrogel. Similarly, the DMSO gel was synthesized by taking the exact amount of the reagents in 1 mL DMSO and heating it at 60 °C for 8 h.

Conversion of G1 to T1:

To the vial containing hydrogel **G$_1$** (synthesized as mentioned above) solid **M** (0.04 mmol, 13.8 mg) was added and the mixture was stirred at 60 °C for 3 h to give a clear light yellow solution. The solution was concentrated and treated with excess acetone to give a faint yellow precipitate. The precipitate was then washed with acetone and dried under vacuum. Yield: 23.5 mg (75%). For the organogel, the same procedure was followed to get the yellow DMSO solution. It was then treated with excess ethyl acetate to get a faint yellow precipitate. Isolated yield: 25 mg (80%). The solid product was dissolved in water and kept for acetone vapor diffusion which allowed the isolation of single crystals suitable for XRD analysis. ^{1}H NMR(D_2O) δ (ppm): 9.14 (s, 4H), 3.04-2.08 (m, 32H). ESI-MS (m/z): 1110.15 [**T1**$(NO_3)_8]^{4+}$ and 1500.21 [**T1**$(NO_3)_9]^{3+}$. Anal. Calcd. For $C_{120}H_{216}N_{84}O_{36}Pd_{12}$: C, 30.74; H, 4.64; N, 25.09; found: C, 30.10; H, 4.85; N, 25.22. **T1** was also synthesized directly by taking a 2 mL aqueous solution of **M** (0.10 mmol, 36.4 mg), to which solid **L^1** (0.05 mmol, 10.7 mg) was added and the mixture was stirred at 50 °C for 4 h. The resulting light-yellow solution was then concentrated and treated with 10 mL acetone to obtain a light yellow precipitate. The precipitate was then washed and dried under reduced pressure. Yield: 31.5 mg (81%).

Conversion of G2 to T2:

Following the above-mentioned procedure, **G2** was treated with M (0.04, 13.8 mg) to get the nanocage **T2** as light yellow powder. Yield: 22.2 mg (73%). This solid was dissolved in water and allowed diffusion of acetone vapor to get single crystals. ^{1}H NMR(D_2O) δ (ppm): 10.05 (s, 3H), 3.04-2.3 (m, 32H). ESI-MS (m/z): 1070.64 [**T2**$(NO_3)_8]^{4+}$ and 1448.53 [**T2**$(NO_3)_9]^{3+}$. Anal. Calcd. For $C_{108}H_{204}N_{84}O_{36}Pd_{12}$: C, 28.62; H, 4.54; N, 25.96; found: C, 28.15; H, 5.08; N, 26.09. T2 was also synthesized by treating 2 mL aqueous solution of M (0.1 mmol, 34.6 mg) with solid **L^2** (0.03 mmol, 9.4 mg) at 60 °C for 3 h. The resulting light

yellow solution was then treated according to the usual method described before to obtain the light yellow product. Yield: 29.2 mg (77%).

Cage (T1) to gel (G1) conversion:

To a 1 mL aqueous solution of **T1** (0.003 mmol, 15.6 mg), $\mathbf{L^1}$ (0.02 mmol, 4.3 mg) was added and the mixture was stirred at 50 °C until all solid $\mathbf{L^1}$ was consumed. Then the solution was heated further at 50 °C for 6 h to obtain the hydrogel. Similar procedure was followed to prepare the organogel with DMSO.

pH monitored self-assembly:

A 5 mL aqueous solution of **M** (0.02 mmol, 6.9 mg) was taken in a 20 mL round bottom flask (pH = 3.60). The solution was then treated with solid $\mathbf{L^1}$ (0.01 mmol, 2.2 mg) at 60 °C for 2 h (pH = 2.36). The change in pH corresponds to the release of two protons per molecule of the ligand.

Gage (T2) to gel (G2) conversion:

1 mL aqueous solution of **T2** (0.003 mmol, 15.1 mg) was added to solid $\mathbf{L^2}$ (0.013 mmol, 3.7 mg) in a clean glass vial and the mixture was stirred at 50 °C until L^1 was consumed. Then the solution was heated at 50 °C for 8 h to obtain the hydrogel. Similar procedure was followed to prepare the organogel in DMSO.

Synthesis of the prism P:

A 2 mL aqueous solution of **M** (0.1 mmol, 34.6 mg) was added to the solid ligand $\mathbf{L^3}$ (0.05 mmol, 10.7 mg) and heated at 55 °C for 2 h to give a light yellow solution. The resulting solution was then concentrated under reduced pressure and treated with 10 mL of acetone to obtain a white precipitate. The precipitate was then washed with acetone and dried under vacuum. Yield: 33 mg (84%). ^{1}H NMR(D_2O) δ (ppm): 10.29 (d, 2H), 8.40 (t, 1H), 7.27 (s, 1H) and 3.32-2.72 (m, 32H). Anal. Calcd. For $C_{60}H_{108}N_{42}O_{18}Pd_6$: C, 30.74; H, 4.64; N, 25.09; found: C, 30.30; H, 4.90; N, 24.89.

Self-sorting experiment:

To a D_2O solution of **M** (0.04 mmol, 13.8 mg), solid ligand $\mathbf{L^1}$ (0.01 mmol, 2.1 mg) and $\mathbf{L^2}$ (0.006 mmol, 1.9 mg) were added and the reaction mixture was heated and stirred at 50 °C

for 6 h. The resulting clear solution was used for ^{1}H NMR spectral analysis. The same solution was further treated with excess KPF_6 salt to get a white precipitate, which was isolated, dried and dissolved in acetonitrile for mass spectral analysis for better ESI-MS result.

Michael addition reactions:

To a solid nitro-alkene **2** (0.02 mmol), 1 mL aqueous solution of the cage **T1** (2 mol %) was added followed by an addition of **3** (0.02 mmol, 3.1 mg). The mixture was stirred at room temperature for the time periods as mentioned in Table 1. Then the reaction mixtures were extracted with chloroform and finally the pure product was obtained from preparative TLC, which was characterized by ^{1}H NMR spectroscopy.

Single crystal XRD structures of T1, T2 and P:

All the cages were crystallized by diffusion of acetone vapour to an aqueous solution of the corresponding cages. Single crystal X-ray data were collected on a Bruker SMART APEX (D8 QUEST) CMOS diffractometer using the SMART/SAINT software.[2] Intensity data were collected using graphite-monochromatized Mo-Kα radiation (0.71073 Å) at 110 K. The structure was solved by direct methods and Fourier analyses and refined by the full-matrix least-squares method based on F^2 with all observed reflections.[3-4] using the SHELX-97[5] program incorporated into WinGX[6]. All non-hydrogen atoms were refined with anisotropic displacement coefficients. The hydrogen atoms bonded to carbon were included in geometric positions and given thermal parameters equivalent to 1.2 times those of the atom to which they were attached. In addition, the structure contains a huge void of disordered solvent molecules and anions, so Squeeze program[7] was applied to account for embedded solvent molecules seriously disordered. Crystallographic data and refinement parameter are given in Table S1.

Table S1: Crystallographic Data and Refinement Parameters of T1, T2 and P.

	**T1**	**T2**	**P**
empirical formula	$C_{120}H_{208}N_{78}O_{52}Pd_{12}$	$C_{120}H_{204}N_{84}O_{47.5}Pd_{12}$	$C_{60}H_{108}N_{42}O_{22}Pd_6$
Fw	4852.43	4860.46	2408.28
T (K)	110(2)	110(2)	110(2)

crystal system	monoclinic	orthorhombic	monoclinic
space group	*C2/c*	*I222*	*P21/c*
a/Å	30.6898(18)	19.9984(8)	18.5319(14)
b/Å	24.2224(15)	22.3102(9)	22.2301(18)
c/Å	33.083(2)	22.5607(9)	28.691(2)
α/deg	90	90	90
β/deg	96.418(2)	90	95.243(2)
γ/deg	90	90	90
V/Å^3	24439(3)	10065.9(7)	11770.5(16)
Z	4	2	4
ρ_{calcd} (g cm^{-3})	1.319	1.604	1.359
μ (Mo Kα) (mm^{-1})	0.934	1.133	0.967
λ/Å	0.71073	0.71073	0.71073
F (000)	9768.0	4888.0	4856.0
collected reflns	293042	153768	302467
unique reflns	16994	11681	20732
GOF (F^2)	0.783	1.066	0.998
R_1[a]	0.0804	0.0433	0.0788
wR_2[b]	0.2500	0.1144	0.2452

[a]$R_1=\Sigma||F_o|-|F_c||/\Sigma|F_o|$, [b]$wR_2=[\Sigma\{w(F_o^2-F_c^2)^2\}/\Sigma\{w(F_o^2)^2\}]^{1/2}$.

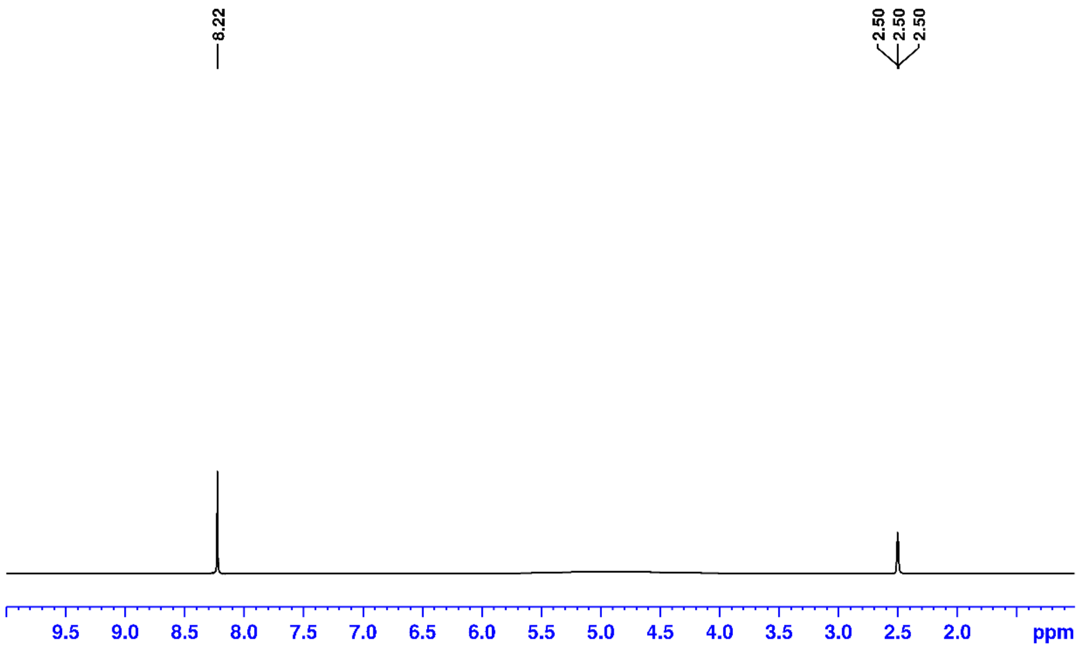

Figure S1: ^{1}H NMR spectra of the linker **L^1** in DMSO-d$_6$.

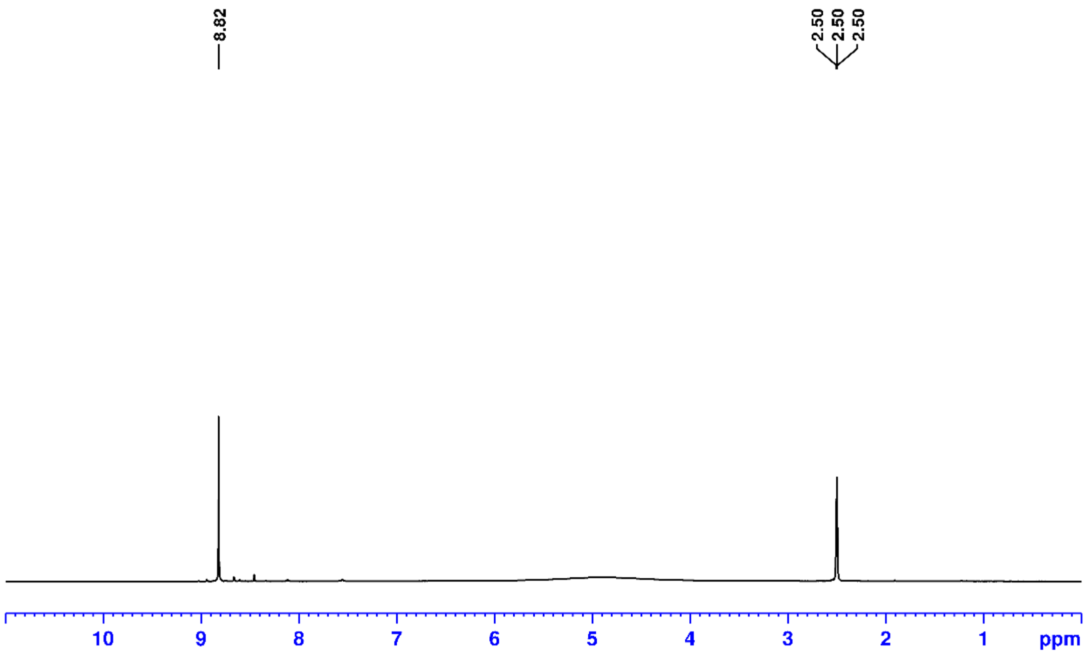

Figure S2: ^{1}H NMR spectra of the linker **L^2** in DMSO-d$_6$.

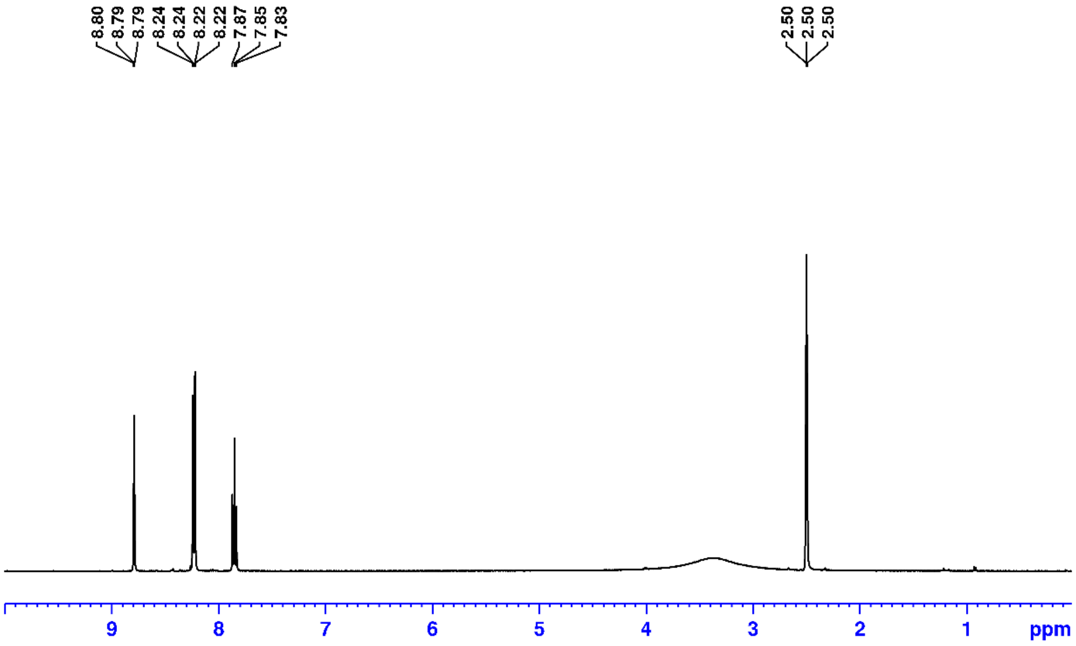

Figure S3: ^{1}H NMR spectra of the linker **L^3** in DMSO-d_6.

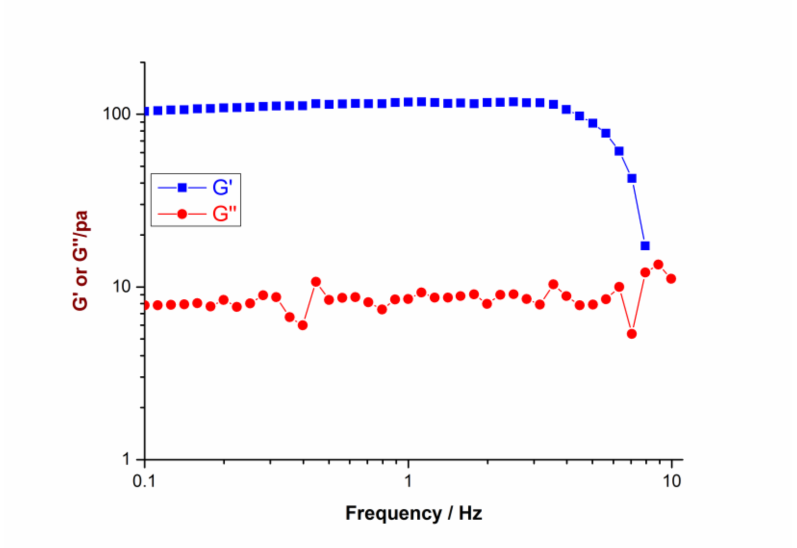

Figure S4: G' and G" vs frequency swap of the hydrogel **G1**.

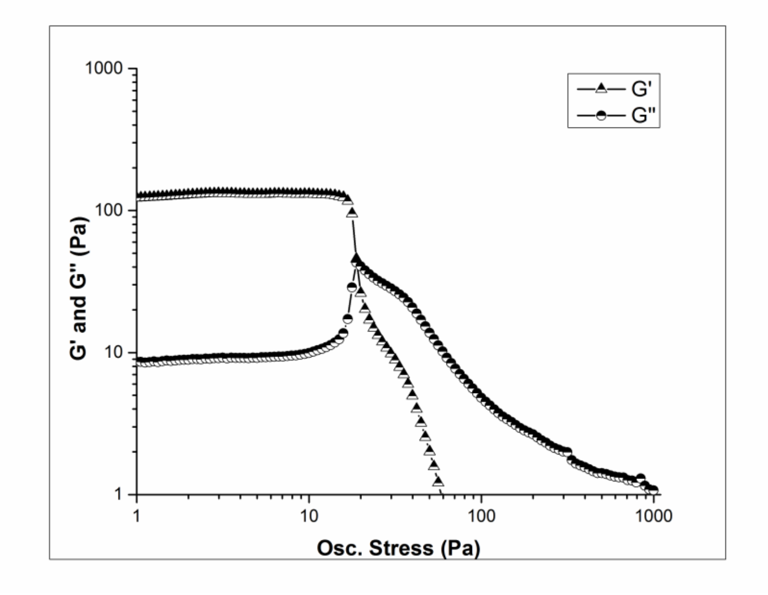

Figure S5: G' and G" vs oscillation stress swap of the DMSO gel **G1**.

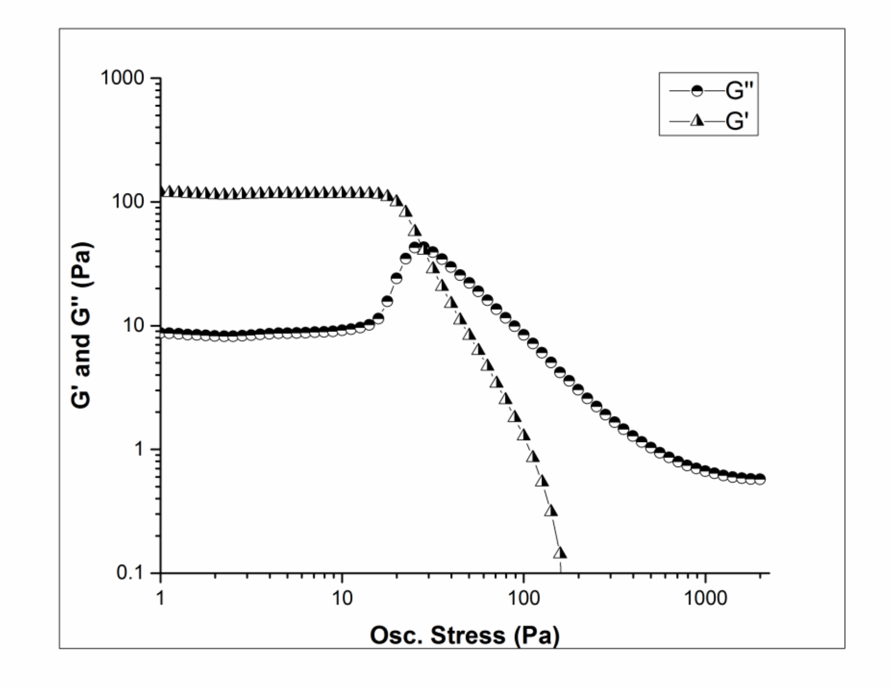

Figure S6: G' and G" vs oscillation stress swap of the hydrogel **G1**.

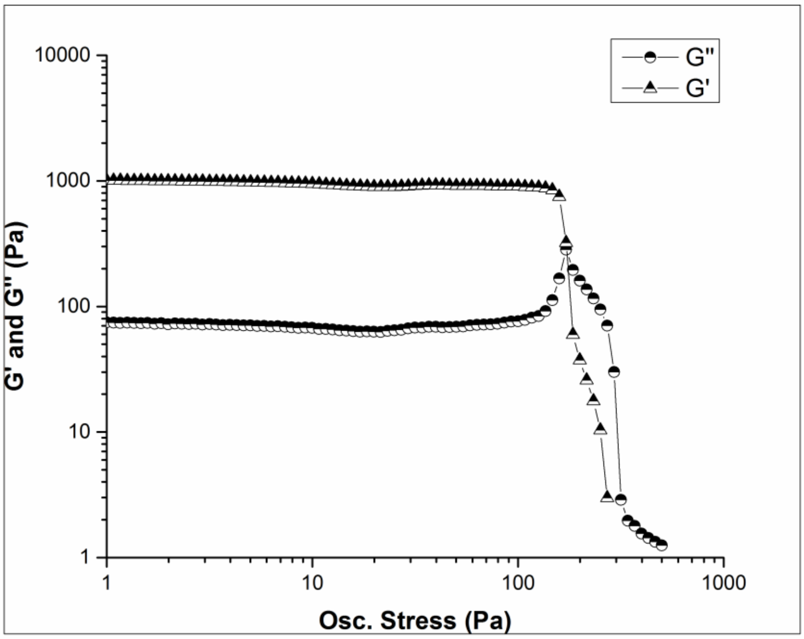

Figure S7: G' and G'' vs oscillation stress swap of the DMDO gel **G2.**

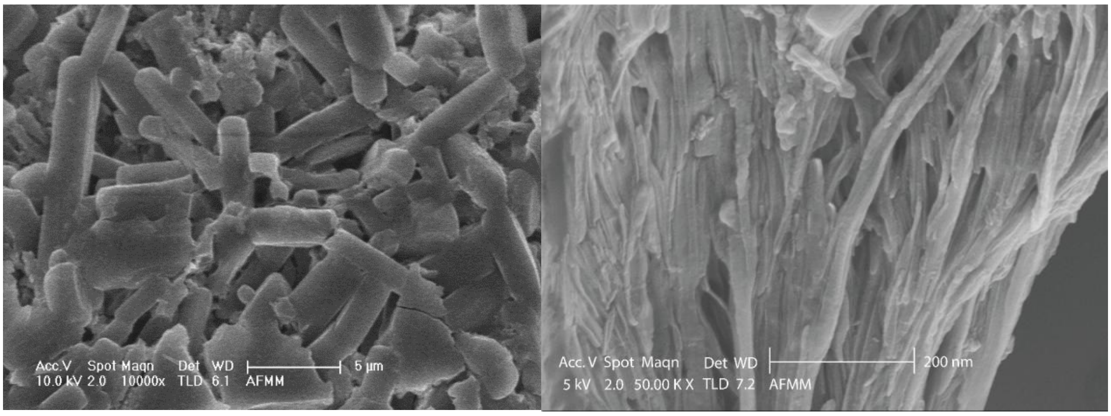

Figure S8: SEM image of the DMSO gel of **G1** (left) and hydrogel of **G2** (right).

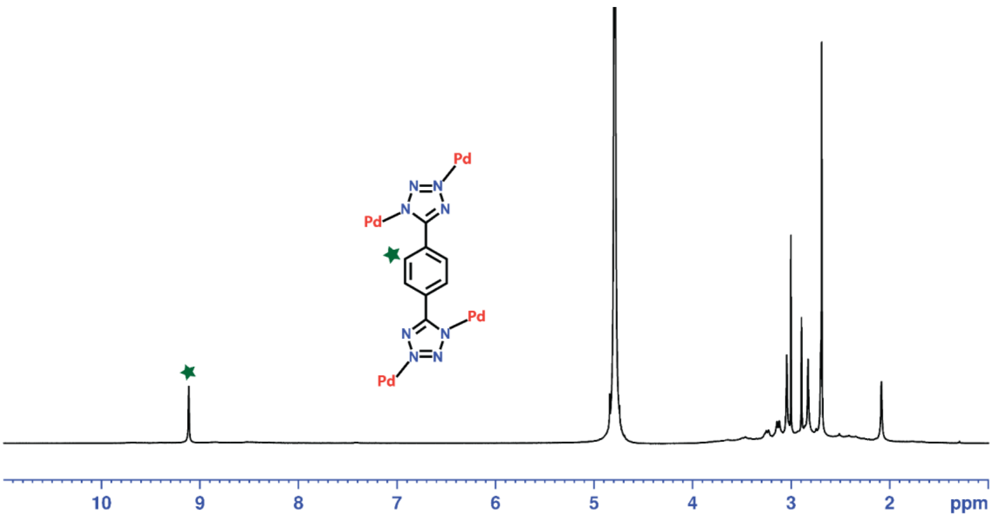

Figure S9: ^{1}H NMR spectra of **T1** in D_2O.

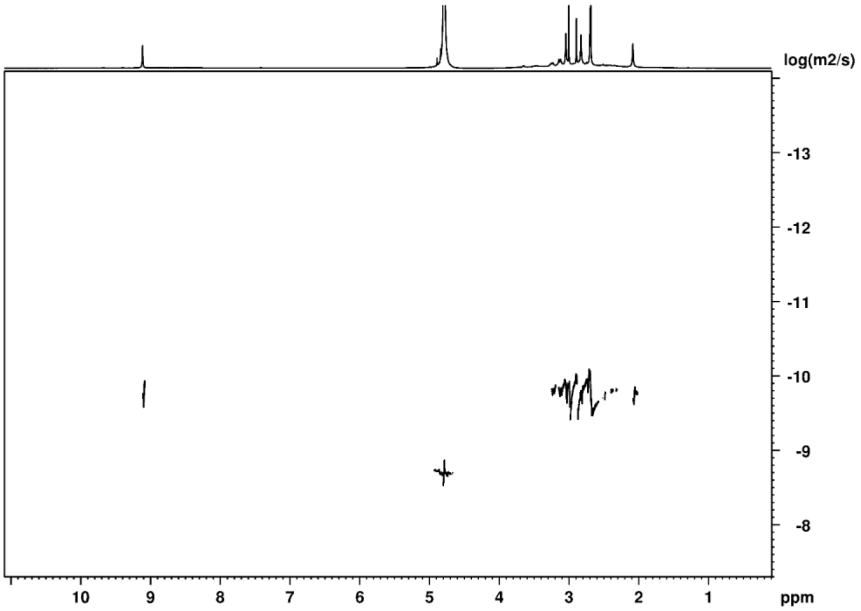

Figure S10: ^{1}H DOSY NMR spectra of **T1** in D_2O.

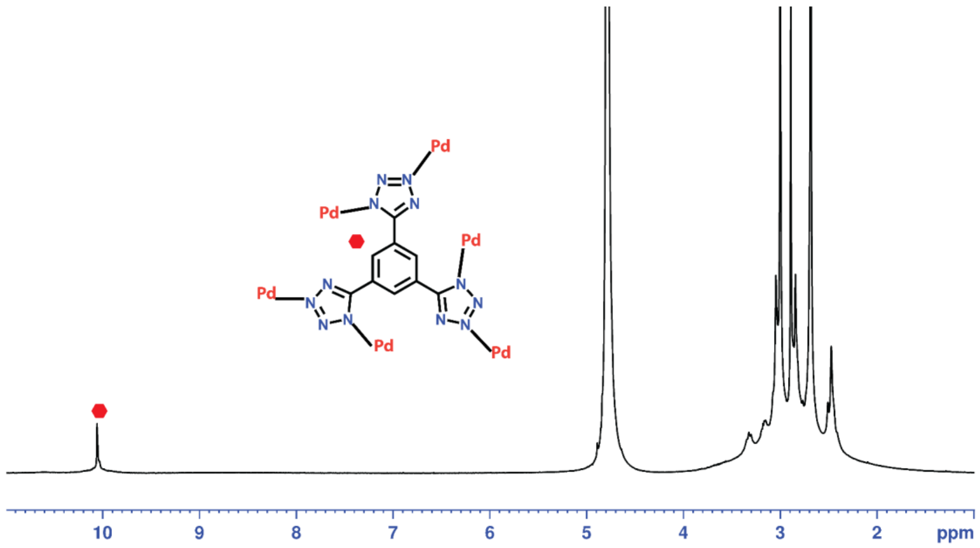

Figure S11: ^{1}H NMR spectra of **T2** in D_2O.

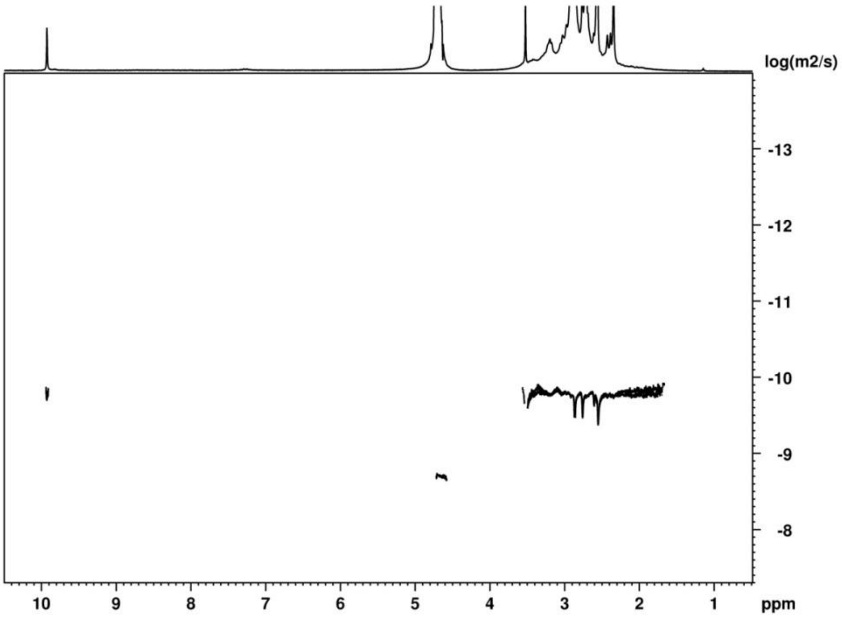

Figure S12: ^{1}H DOSY NMR spectra of **T2** in D_2O.

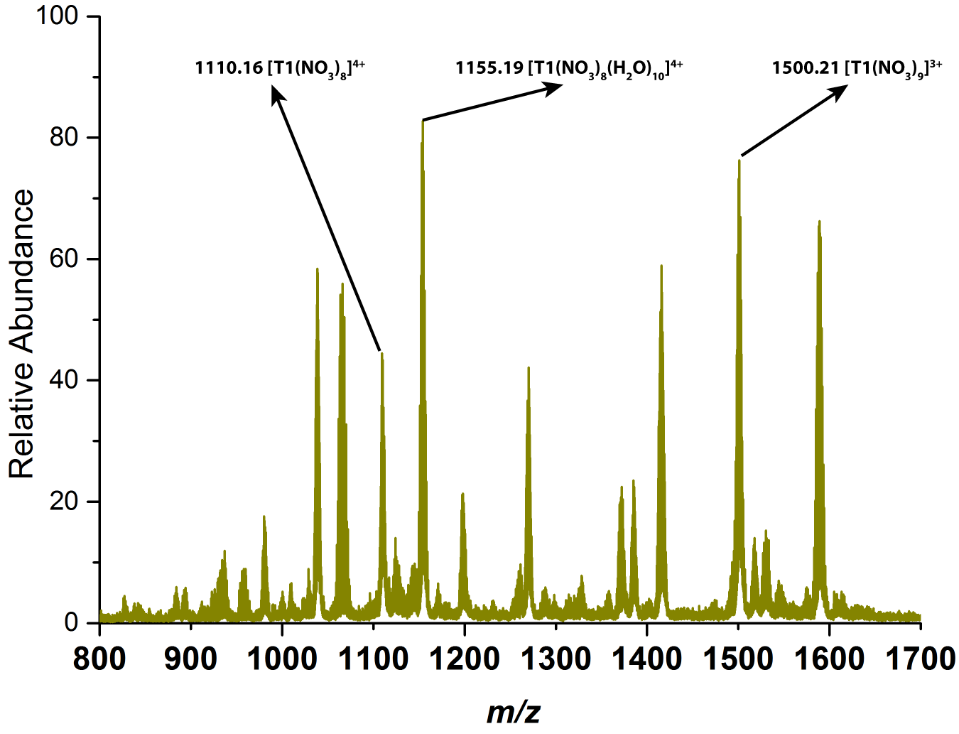

Figure S13: ESI-MS spectra of **T1** in H_2O.

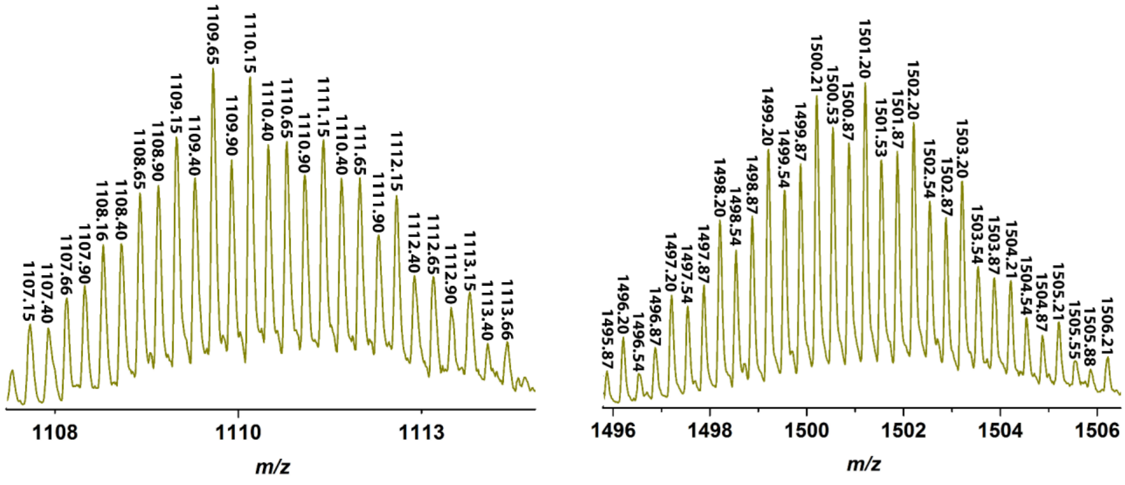

[**T1**$(NO_3)_8]^{4+}$ [**T1**$(NO_3)_9]^{3+}$

Figure S14: Experimental isotropic patterns of the fragments [**T1**$(NO_3)_8]^{4+}$ (left) and [**T1**$(NO_3)_9]^{3+}$ (right) for the cage **T1**.

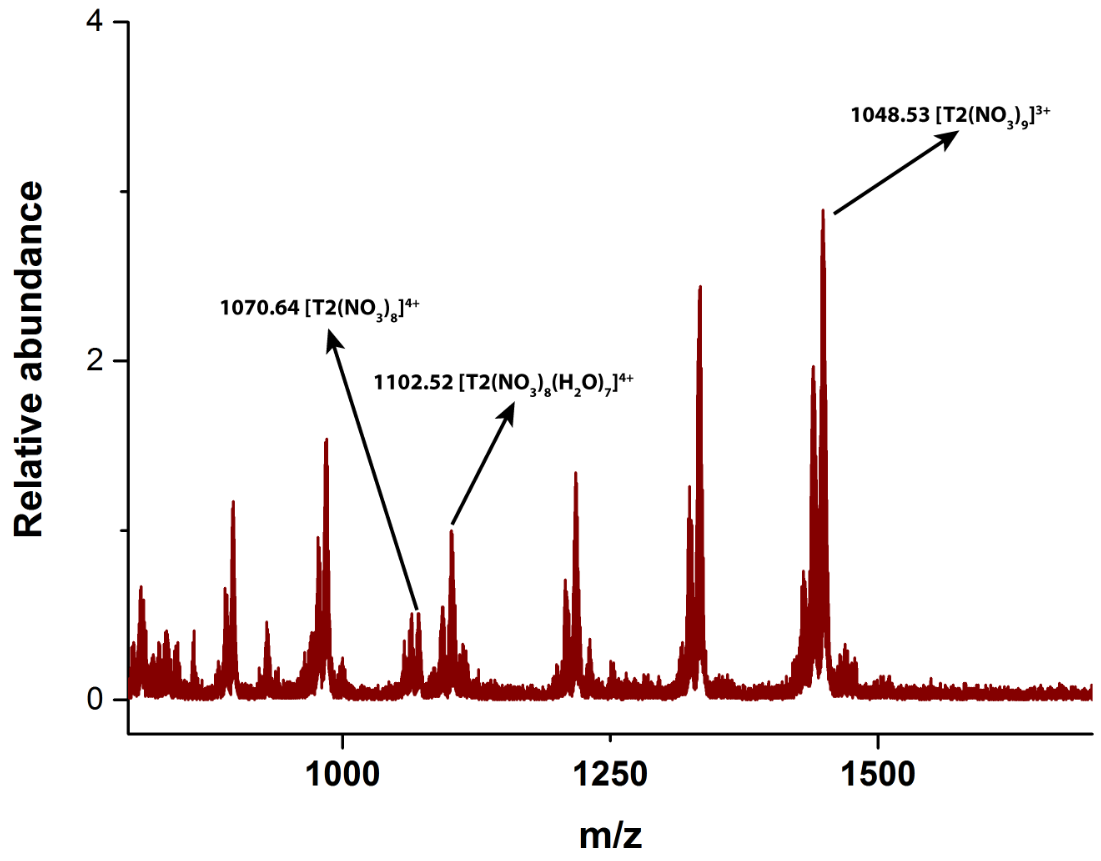

Figure S15: ESI-MS spectra of **T2** in H_2O.

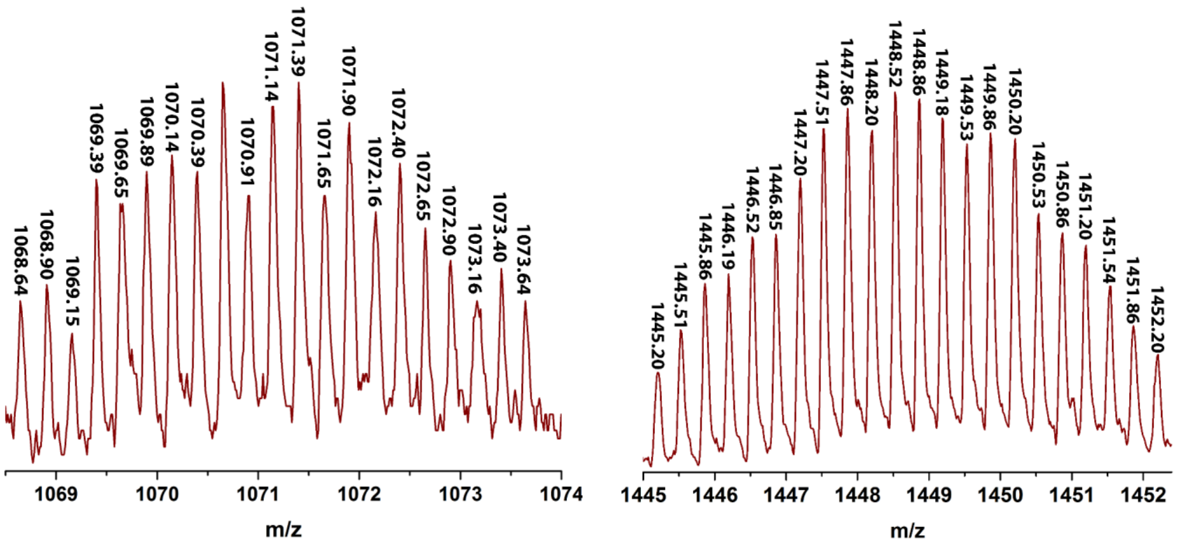

[**T2**(NO_3)$_8$]$^{4+}$ [**T2**(NO_3)$_9$]$^{3+}$

Figure S16: Experimental isotropic patterns of the fragments [**T2**(NO_3)$_8$]$^{4+}$ (left) and [**T2**(NO_3)$_9$]$^{3+}$ (right).

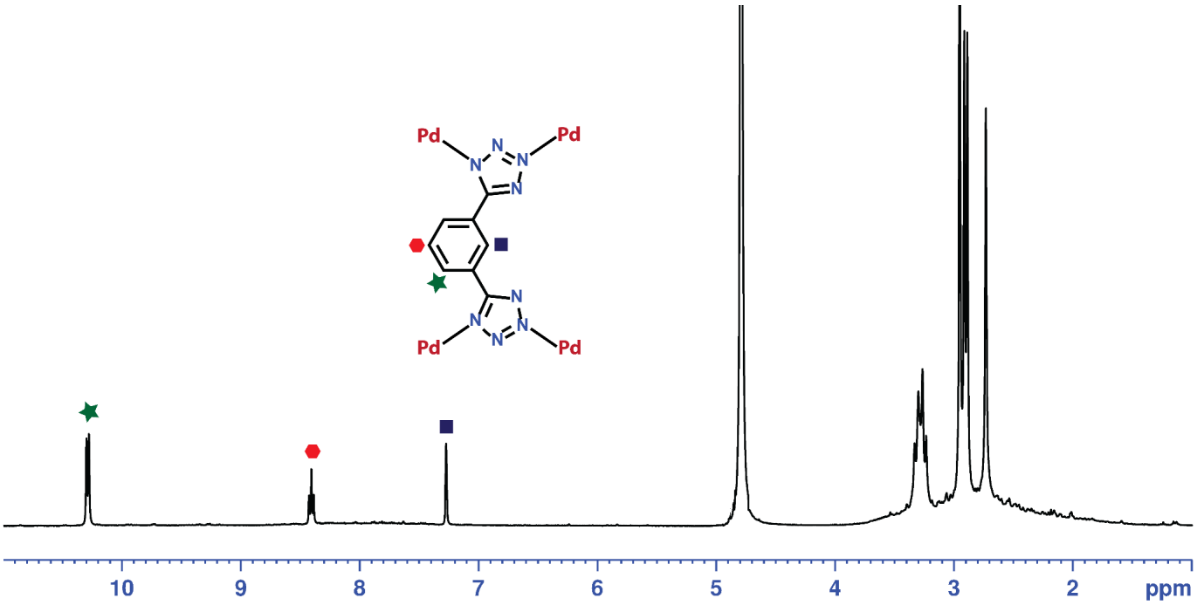

Figure S17: ^{1}H NMR spectra of **P** in D_2O.

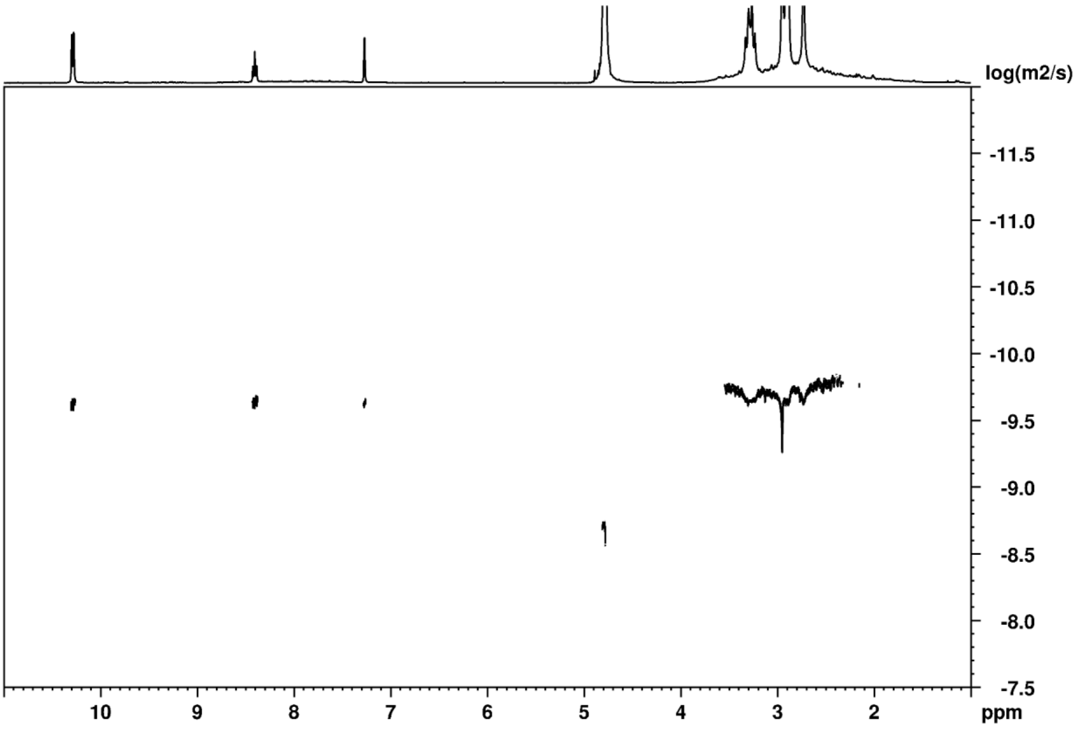

Figure S18: ^{1}H DOSY NMR of **P** in D_2O.

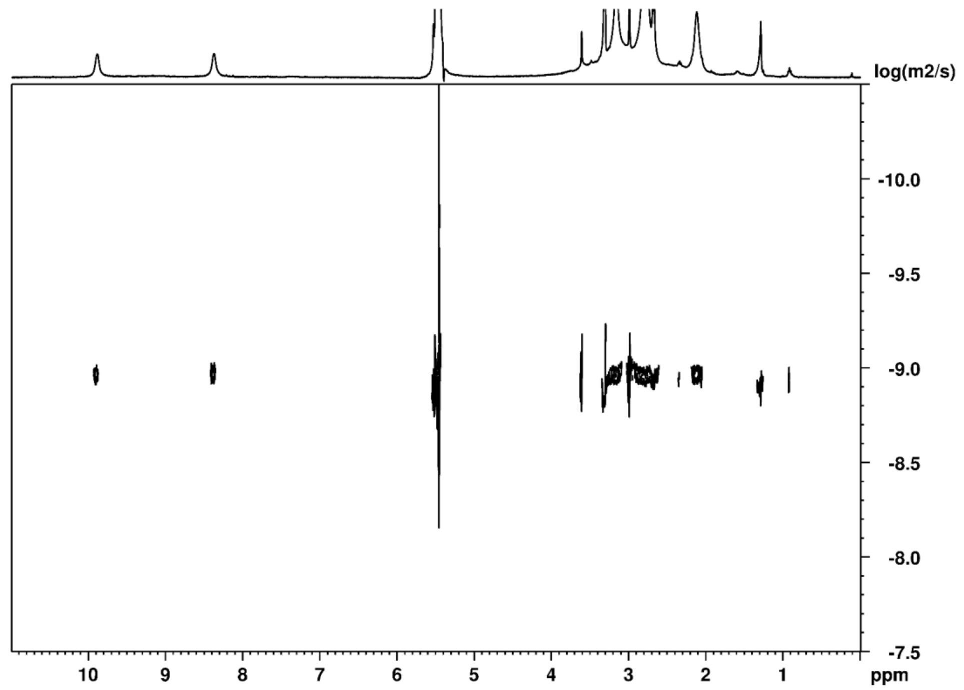

Figure S19: ^{1}H DOSY NMR spectra of the nano-cage **T1** in D_2O at -45 °C.

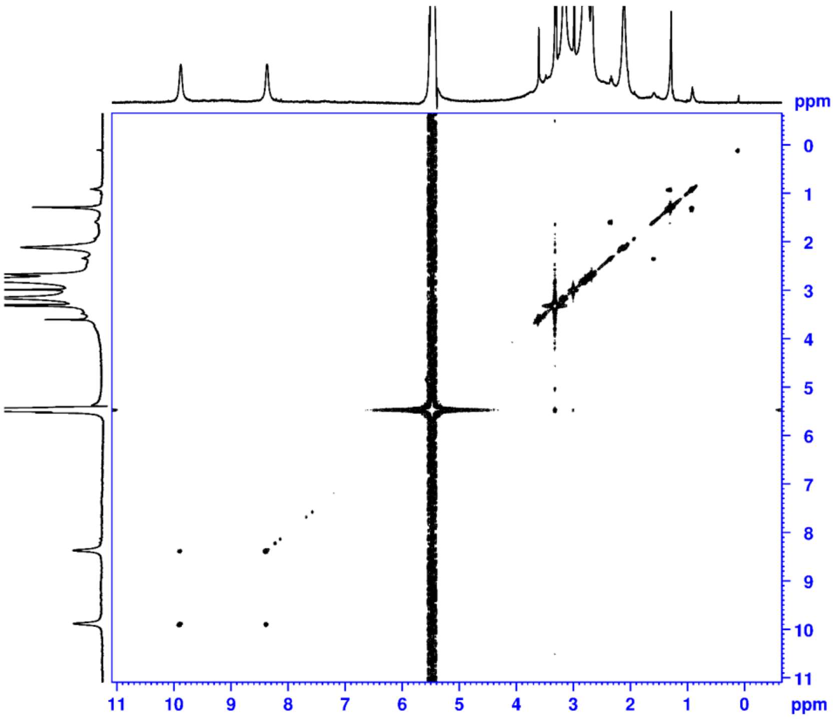

Figure S20: ^{1}H COSY NMR spectra of the nanocage **T1** in D_2O at -45 °C.

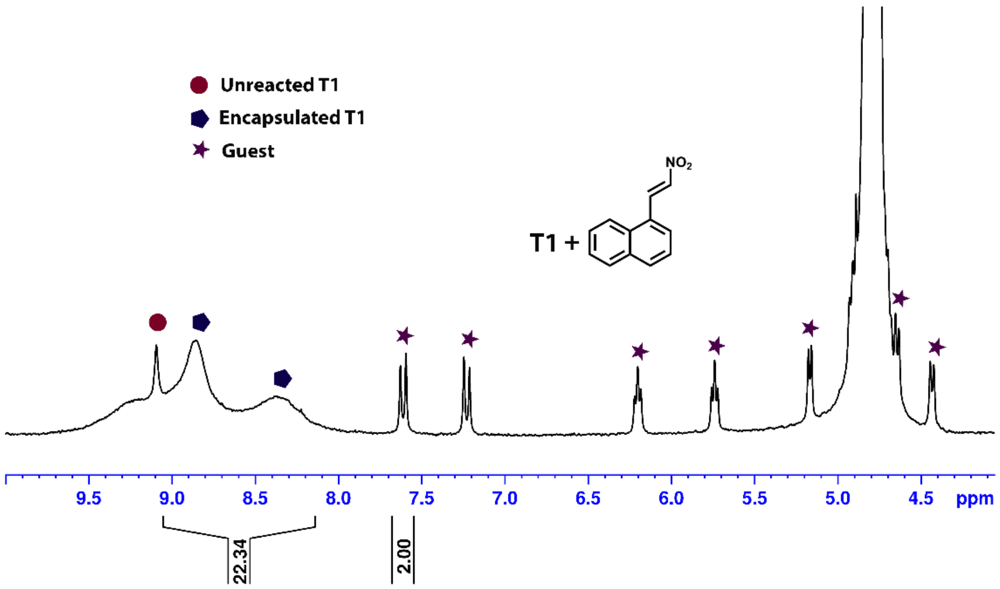

Figure S21: ^{1}H NMR spectroscopy of guest (**1**) encapsulated **T1** in D_2O.

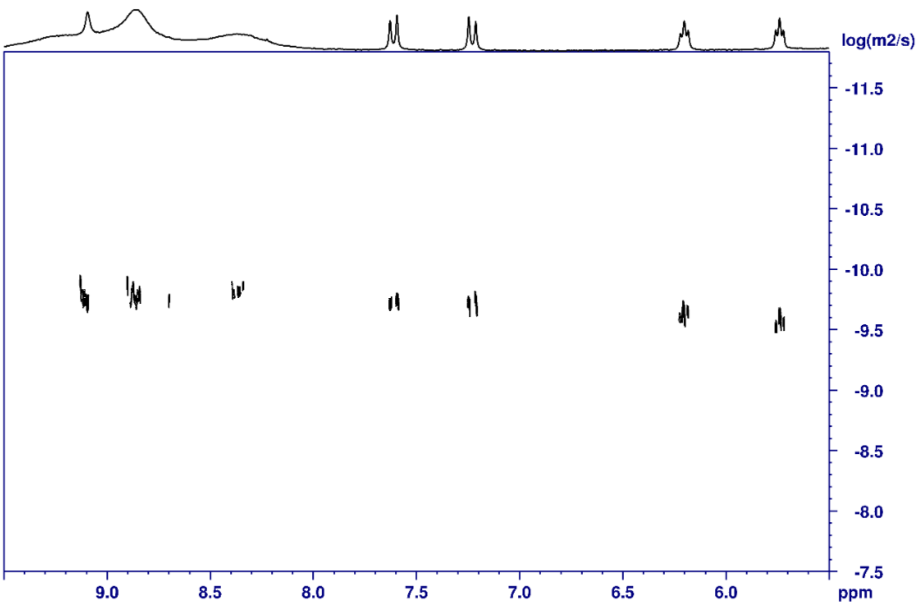

Figure S22: ^{1}H DOSY NMR spectra of guest (**1**) encapsulated **T1** in D_2O.

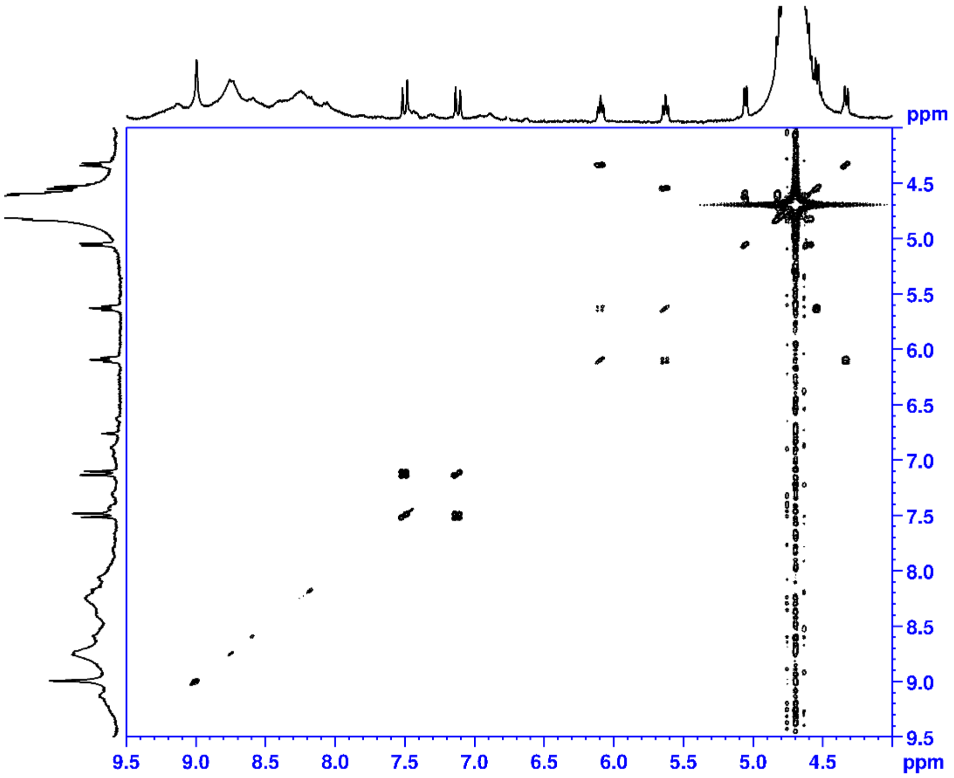

Figure S23: ^{1}H COSY NMR spectra of guest (**1**) encapsulated **T1** in D_2O.

^{1}H NMR spectra of the Michael reaction products:

1,3-Dimethyl-5-(2-nitro-1-pyren-1-yl-ethyl)-pyrimidine-2,4,6-trione (3a):

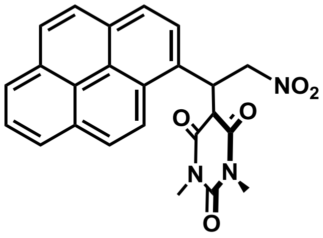

^{1}H NMR (400 MHz, $CDCl_3$) δ (ppm): 8.32 (d, *J*=8.0 Hz, 1H, ArH), 8.24-8.19 (m, 1H, ArH), 8.12-8.09 (m, 2H, ArH), 8.07-8.00 (m, 1H, ArH), 7.72 (d, *J*=8.0 Hz, 1H, ArH), 5.45-5.39 (m, 1H, CH), 5.59-5.54 (m, 1H, CH), 5.22-5.17 (m, 1H, CH), 4.06 (d, *J*=4.0 Hz, 1H, CH), 3.14 (s, 3H, NCH_3), 2.69 (s, 3H, NCH_3).

1,3-Dimethyl-5-(1-naphthalen-1-yl-2-nitro-ethyl)-pyrimidine-2,4,6-trione (4b):

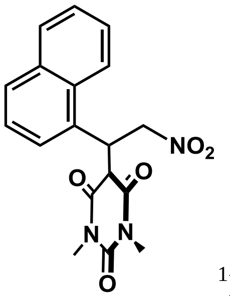

^{1}H NMR (400 MHz, $CDCl_3$) δ (ppm): 7.39-7.35 (m, 1H, ArH), 7.84-7.81 (m, 2H, ArH), 7.62-7.58 (m, 1H, ArH), 7.54-7.50 (m, 1H, ArH), 7.42-7.38 (m, 1H, ArH), 7.26-7.22 (m, 1H, ArH), 5.45-5.39 (m, 2H, 2 X CH), 5.07 (dd, *J*=12.0 Hz, *J*=8.0 Hz, 1H, CH), 3.95 (d, *J*=4.0 Hz, 1H, CH), 3.12 (s, 3H, NCH_3), 2.72 (s, 3H, NCH_3).

1,3-Dimethyl-5-(1-methyl-2-nitro-1-phenyl-ethyl)-pyrimidine-2,4,6-trione (4c):

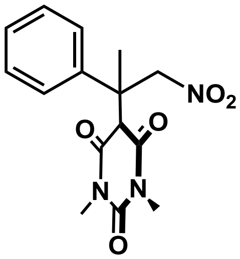

^{1}H NMR (400 MHz, $CDCl_3$) δ (ppm): 7.39-7.35 (m, 3H, ArH), 7.10-7.07 (m, 2H, ArH), 5.36 (d, *J*=12.0 Hz, 1H, CH), 5.00 (d, *J*=12.0 Hz, 1H, CH), 4.05 (s, 1H, CH), 3.04 (s, 3H, NCH_3), 3.02 (s, 3H, NCH_3), 3.22 (s, 3H, CH_3).

1,3-Dimethyl-5-(2-nitro-1-p-tolyl-ethyl)-pyrimidine-2,4,6-trione (4d):

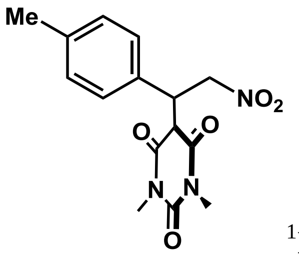

^{1}H NMR (400 MHz, $CDCl_3$) δ (ppm): 7.08 (d, *J*=8.0 Hz, 2H, ArH), 6.91 (d, *J*=8.0 Hz, 2H, ArH), 5.29-5.23 (m, 1H, CH), 5.00-4.95 (m, 1H, CH), 4.48-4.44 (m, 1H, CH), 3.83 (d, *J*=4.0 Hz, 1H, CH), 3.15 (s, 3H, NCH_3), 3.09 (s, 3H, NCH_3), 2.29 (s, 3H, CH_3).

5-[1-(4-Methoxy-phenyl)-2-nitro-ethyl]-1,3-dimethyl-pyrimidine-2,4,6-trione (4e):

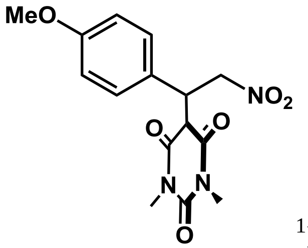

^{1}H NMR (400 MHz, $CDCl_3$) δ (ppm): 6.96 (d, *J*=8.0 Hz, 2H, ArH), 6.79 (d, *J*=8.0 Hz, 2H, ArH), 5.27-5.21 (m, 1H, CH), 4.99-4.94 (m, 1H, CH), 4.48-4.43 (m, 1H, CH), 3.83 (d, *J*=4.0 Hz, 1H, CH), 3.76 (s, 3H, OCH_3), 3.15 (s, 3H, NCH_3), 3.10 (s, 3H, NCH_3).

5-(1-Furan-2-yl-2-nitro-ethyl)-1,3-dimethyl-pyrimidine-2,4,6-trione (4f):

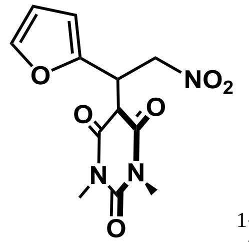

^{1}H NMR (400 MHz, $CDCl_3$) δ (ppm): 7.29 (d, *J*=8.0 Hz, 1H, ArH), 7.29-6.30 (m, 1H, ArH), 6.16 (d, *J*=4.0 Hz, 1H, ArH), 5.20-5.15 (m, 1H, CH), 4.99-4.94 (m, 1H, CH), 4.71-4.67 (m, 1H, CH), 3.84 (d, *J*=4.0 Hz, 1H, CH), 3.22 (s, 6H, NCH_3).

Coordinates of the optimized geometry of 1⊂T1:

Centre number	Atomic Number	Atomic Type	Coordinates (Angstrom)		
			X	Y	Z
1	7	0	4.0429	0.0028	-8.9717
2	7	0	6.1518	1.1496	-7.5002
3	6	0	4.8205	1.1387	-9.595
4	6	0	5.8946	2.5209	-6.9557
5	1	0	5.7361	2.47	-5.8629
6	1	0	4.9791	2.9473	-7.4049
7	1	0	6.7182	3.2373	-7.146
8	6	0	7.415	0.5795	-6.932
9	1	0	7.4748	0.7478	-5.8444
10	1	0	8.3381	1.006	-7.3755
11	1	0	7.4322	-0.5166	-7.1027

12	6	0	2.5746	0.2407	-9.1452
13	1	0	1.9905	-0.6537	-8.8695
14	1	0	2.2858	0.501	-10.1837
15	1	0	2.2488	1.0639	-8.4815
16	6	0	4.4428	-1.3118	-9.5676
17	1	0	5.5349	-1.4691	-9.4678
18	1	0	4.2007	-1.4101	-10.645
19	1	0	3.9518	-2.1413	-9.0275
20	6	0	6.2345	1.1706	-9.0083
21	46	0	4.5564	-0.0364	-6.9913
22	1	0	4.8823	1.0554	-10.713
23	1	0	4.2909	2.104	-9.4098
24	1	0	6.7882	2.063	-9.4012
25	1	0	6.8317	0.2958	-9.3708
26	7	0	8.8294	-2.2416	-2.5284
27	6	0	8.7798	-1.4182	-1.2805
28	1	0	8.4876	-2.0423	-0.4154
29	1	0	8.0083	-0.6231	-1.3619
30	1	0	9.7355	-0.9196	-1.0308
31	6	0	7.2634	-5.6627	-1.0735
32	1	0	6.3387	-6.1043	-1.4833
33	1	0	6.9744	-4.9756	-0.2567
34	1	0	7.8447	-6.497	-0.6318
35	6	0	9.3589	-4.4855	-1.599
36	1	0	9.2688	-4.1948	-0.5248
37	1	0	10.1078	-5.3211	-1.6107
38	6	0	9.0982	-1.3811	-3.7251
39	1	0	8.8384	-1.9374	-4.6469
40	1	0	10.1569	-1.0596	-3.8117
41	1	0	8.4923	-0.4607	-3.6962
42	6	0	8.1552	-5.7395	-3.3683
43	1	0	8.7352	-5.193	-4.1378
44	1	0	7.1625	-5.9495	-3.8056
45	1	0	8.669	-6.7082	-3.205
46	6	0	9.8877	-3.3159	-2.4339
47	1	0	10.1716	-3.6608	-3.46
48	1	0	10.846	-2.939	-1.9901
49	46	0	7.0267	-3.1968	-2.6883
50	7	0	8.0096	-4.9087	-2.1301
51	6	0	2.0673	-7.23	-8.1587
52	1	0	1.3591	-7.7205	-8.8766
53	1	0	3.0345	-7.1783	-8.7203
54	6	0	2.2175	-8.0967	-6.9058
55	1	0	2.6446	-9.0955	-7.1919
56	1	0	1.2205	-8.3309	-6.461

57	6	0	4.5218	-7.4441	-6.2581
58	1	0	4.6875	-7.0538	-7.281
59	1	0	4.9429	-8.47	-6.2335
60	1	0	5.1216	-6.814	-5.5759
61	6	0	0.1454	-5.7844	-7.5496
62	1	0	-0.1671	-6.5371	-6.8026
63	1	0	-0.4411	-5.9741	-8.4706
64	1	0	-0.1435	-4.79	-7.1629
65	6	0	2.8388	-7.9552	-4.5306
66	1	0	1.8453	-7.6461	-4.1549
67	1	0	3.5913	-7.597	-3.8071
68	1	0	2.8857	-9.0636	-4.5077
69	6	0	2.0578	-4.8536	-8.7967
70	1	0	1.582	-3.8726	-8.634
71	1	0	1.8158	-5.1622	-9.8346
72	1	0	3.1556	-4.7104	-8.7329
73	46	0	2.5216	-5.4089	-5.9628
74	7	0	1.6274	-5.8419	-7.757
75	7	0	3.0715	-7.3731	-5.8906
76	6	0	-6.3949	-6.1217	-2.5517
77	1	0	-5.6804	-6.0993	-1.7065
78	1	0	-5.941	-5.5456	-3.3764
79	1	0	-6.479	-7.1691	-2.9045
80	6	0	-8.5502	-5.1976	-3.3157
81	1	0	-9.5234	-4.7767	-2.9948
82	1	0	-8.774	-6.0701	-3.9623
83	1	0	-8.0587	-4.4294	-3.9379
84	6	0	-8.0436	-4.9067	1.518
85	1	0	-7.49	-4.0422	1.9272
86	1	0	-7.3059	-5.6987	1.2937
87	1	0	-8.7059	-5.2924	2.3187
88	6	0	-8.4163	-6.4748	-1.1959
89	1	0	-8.9309	-7.3081	-1.7448
90	1	0	-7.6758	-6.985	-0.534
91	6	0	-9.7983	-3.4553	0.5737
92	1	0	-9.3849	-2.671	1.2283
93	1	0	-10.7076	-3.8462	1.0754
94	1	0	-10.1158	-2.971	-0.3724
95	6	0	-9.4444	-5.6991	-0.3684
96	1	0	-9.924	-6.3833	0.3795
97	1	0	-10.2936	-5.3575	-1.0131
98	46	0	-7.4081	-3.787	-1.08
99	7	0	-7.7001	-5.5237	-2.1264
100	7	0	-8.7745	-4.5071	0.2736

101	6	0	-9.4449	3.7019	-2.6495
102	1	0	-8.4865	3.8592	-3.1703
103	1	0	-10.1553	4.4562	-3.0465
104	1	0	-9.8229	2.6986	-2.934
105	6	0	-9.3043	1.5972	2.249
106	1	0	-8.4122	2.2182	2.4543
107	1	0	-8.9888	0.5412	2.3051
108	1	0	-10.0299	1.7538	3.0732
109	6	0	-10.6204	3.8388	-0.4924
110	1	0	-11.0339	4.8808	-0.4655
111	1	0	-11.3746	3.2597	-1.0835
112	6	0	-10.8173	0.8768	0.4407
113	1	0	-11.2421	1.138	-0.5484
114	1	0	-11.6736	0.7239	1.1286
115	1	0	-10.3004	-0.092	0.3189
116	6	0	-8.4101	4.9254	-0.7766
117	1	0	-7.3991	4.8099	-1.2074
118	1	0	-8.303	4.9755	0.3222
119	1	0	-8.8098	5.9037	-1.1114
120	6	0	-10.5123	3.2873	0.9316
121	1	0	-11.5334	3.2572	1.3985
122	1	0	-9.9225	3.9762	1.5826
123	46	0	-8.3611	2.0415	-0.5004
124	7	0	-9.269	3.7611	-1.163
125	7	0	-9.8524	1.9284	0.8954
126	6	0	-8.1204	2.0703	-6.1311
127	1	0	-7.7269	2.7813	-5.3869
128	1	0	-8.7833	1.3564	-5.6012
129	1	0	-8.7485	2.6586	-6.832
130	46	0	-6.2594	-0.1269	-5.5546
131	7	0	-6.4539	-1.4246	-7.132
132	7	0	-7.0129	1.3166	-6.8021
133	6	0	-7.6628	-2.2654	-6.8571
134	1	0	-8.5633	-1.6284	-6.7524
135	1	0	-7.5425	-2.8048	-5.9005
136	1	0	-7.8841	-3.0111	-7.6467
137	6	0	-5.8857	2.2357	-7.1598

138	1	0	-5.0936	1.6853	-7.6984
139	1	0	-5.4295	2.6524	-6.2415
140	1	0	-6.1892	3.0862	-7.8024
141	6	0	-7.5505	0.6142	-8.0268
142	1	0	-8.6019	0.2765	-7.8421
143	1	0	-7.631	1.294	-8.9154
144	6	0	-5.2391	-2.2842	-7.2974
145	1	0	-4.3302	-1.6555	-7.2778
146	1	0	-5.2349	-2.8678	-8.2396
147	1	0	-5.1577	-3.0133	-6.4722
148	6	0	-6.6535	-0.5789	-8.3679
149	1	0	-5.6645	-0.2339	-8.7531
150	1	0	-7.1087	-1.1557	-9.2164
151	7	0	-0.499	7.6678	1.1342
152	7	0	-5.1457	2.058	-3.5723
153	6	0	-4.6798	3.6276	-1.6442
154	7	0	-1.6617	5.961	1.8841
155	7	0	-6.1426	1.2245	-3.8972
156	7	0	-0.7704	6.9098	2.206
157	7	0	-6.774	2.1484	-2.0078
158	7	0	-1.1872	7.228	0.0742
159	6	0	-5.513	2.6634	-2.3687
160	6	0	-1.9549	6.1386	0.5309
161	6	0	-3.6459	4.314	-2.3138
162	1	0	-3.5134	4.1698	-3.3899
163	6	0	-2.9102	5.3248	-0.2276
164	7	0	-7.114	1.2883	-2.9745
165	6	0	-2.7733	5.1541	-1.6169
166	1	0	-1.9647	5.6706	-2.1481
167	6	0	-3.9719	4.6758	0.4344
168	1	0	-4.1011	4.8148	1.5112
169	6	0	-4.8497	3.8417	-0.2638
170	1	0	-5.6609	3.3295	0.2671
171	7	0	6.0213	-1.4298	-3.3881
172	7	0	5.6101	-0.362	-2.6913
173	7	0	5.7346	-1.266	-4.6868
174	7	0	5.1159	-0.0931	-4.8788
175	6	0	4.4777	1.8212	-3.2484
176	6	0	5.0346	0.5146	-3.6133
177	6	0	5.0823	2.5781	-2.2249
178	1	0	6.0043	2.213	-1.7527
179	6	0	3.3174	2.3176	-3.8664
180	1	0	2.8556	1.7529	-4.6834
181	7	0	1.219	7.0906	-1.6528
182	7	0	1.4288	5.9347	-2.2976
183	7	0	2.2773	7.3881	-0.8849

184	7	0	3.2048	6.4301	-0.9914
185	6	0	3.3276	4.2557	-2.3811
186	6	0	2.69	5.4852	-1.902
187	6	0	2.7495	3.5199	-3.435
188	1	0	1.8401	3.8904	-3.9164
189	6	0	4.5139	3.7784	-1.793
190	1	0	4.9842	4.3464	-0.9798
191	7	0	-7.0822	-1.9604	0.0328
192	7	0	-7.8171	-0.9606	-0.4677
193	7	0	-7.4807	0.1951	0.1222
194	7	0	-6.5275	-0.0162	1.0417
195	6	0	-4.8941	-1.4889	3.1031
196	1	0	-5.3479	-0.548	3.4252
197	6	0	-5.2832	-2.0641	1.8769
198	6	0	-6.2531	-1.3842	1.0145
199	6	0	-3.9263	-2.0998	3.9052
200	1	0	-3.6316	-1.6398	4.8549
201	7	0	-1.9253	-3.785	5.6286
202	7	0	-0.8555	-4.5604	5.8465
203	7	0	-0.4528	-5.1259	4.6997
204	7	0	-1.2555	-4.7461	3.6968
205	6	0	-3.7388	-3.8944	2.2886
206	1	0	-3.2956	-4.8494	1.971
207	6	0	-3.3112	-3.2946	3.49
208	6	0	-2.2164	-3.9002	4.2557
209	6	0	-4.7121	-3.29	1.4902
210	1	0	-5.0264	-3.7658	0.5509
211	7	0	-6.0769	-2.9746	-2.5538
212	7	0	-4.764	-3.1946	-2.7181
213	7	0	2.9429	-1.3413	-6.4713
214	7	0	3.0731	-2.675	-6.4883
215	7	0	-6.5098	-2.0841	-3.4568
216	7	0	1.9481	-3.2546	-6.0509
217	6	0	-2.9946	-2.3498	-4.3218
218	6	0	-0.3335	-2.2794	-5.2925
219	6	0	-2.1299	-3.448	-4.1405
220	1	0	-2.4861	-4.3315	-3.6041
221	7	0	1.7197	-1.006	-6.0377
222	7	0	-5.4933	-1.6809	-4.2286
223	6	0	-4.3586	-2.3895	-3.7831
224	6	0	-0.8181	-3.4167	-4.6228
225	1	0	-0.156	-4.2737	-4.4644
226	6	0	1.0537	-2.2035	-5.7652
227	6	0	-2.5175	-1.2206	-5.011
228	1	0	-3.1778	-0.3584	-5.1578
229	6	0	-1.2036	-1.1866	-5.4856

230	1	0	-0.8409	-0.2942	-6.0023
231	7	0	6.325	7.1509	1.7669
232	7	0	5.9765	7.7865	-0.9576
233	6	0	7.5361	7.5543	0.9581
234	6	0	6.5173	6.7891	-1.9347
235	1	0	5.6885	6.266	-2.4443
236	1	0	7.1278	6.0302	-1.4131
237	1	0	7.159	7.2388	-2.7201
238	6	0	5.1554	8.8237	-1.6613
239	1	0	4.4756	8.3629	-2.3962
240	1	0	5.7596	9.5756	-2.21
241	1	0	4.5323	9.3648	-0.9197
242	6	0	6.632	5.9123	2.5501
243	1	0	5.829	5.6875	3.2735
244	1	0	7.5746	5.9756	3.1307
245	1	0	6.709	5.0459	1.867
246	6	0	5.8955	8.2621	2.6748
247	1	0	5.6796	9.1788	2.0912
248	1	0	6.6516	8.5366	3.4383
249	1	0	4.964	7.9899	3.2009
250	6	0	7.096	8.4574	-0.1964
251	46	0	4.8102	6.8188	0.4308
252	1	0	8.3089	8.0861	1.5754
253	1	0	8.0574	6.6437	0.5761
254	1	0	7.978	8.7013	-0.8452
255	1	0	6.7546	9.4521	0.1875
256	7	0	0.1064	9.3821	-3.3248
257	6	0	-0.0676	8.4966	-4.52
258	1	0	-1.1332	8.2427	-4.6631
259	1	0	0.481	7.5467	-4.3721
260	1	0	0.2904	8.9485	-5.4671
261	6	0	-3.4346	8.8115	-1.4675
262	1	0	-3.4904	8.4013	-0.4441
263	1	0	-3.4786	7.9614	-2.1723
264	1	0	-4.3455	9.4266	-1.6142
265	6	0	-2.19	10.2764	-3.0047
266	1	0	-2.6862	9.6262	-3.7641
267	1	0	-2.805	11.2156	-2.985
268	6	0	1.5403	9.7876	-3.1695
269	1	0	1.7078	10.1645	-2.1403
270	1	0	1.8579	10.5828	-3.8761
271	1	0	2.2172	8.9332	-3.3317
272	6	0	-1.9494	10.5498	-0.5518
273	1	0	-1.0175	11.1273	-0.7122
274	1	0	-1.8322	10.0136	0.4068
275	1	0	-2.7692	11.2871	-0.4346

276	6	0	-0.759	10.616	-3.4301
277	1	0	-0.3398	11.4319	-2.7881
278	1	0	-0.7649	11.0536	-4.4632
279	46	0	-0.53	8.332	-1.6844
280	7	0	-2.1613	9.5732	-1.6678
281	6	0	0.4778	8.8442	6.4804
282	1	0	0.6793	8.9102	7.5816
283	1	0	0.8353	9.8257	6.0772
284	6	0	-1.0243	8.6764	6.2374
285	1	0	-1.5709	9.5655	6.6522
286	1	0	-1.4254	7.804	6.8067
287	6	0	-1.1792	9.7815	4.0242
288	1	0	-0.1962	10.26	4.2026
289	1	0	-1.954	10.5204	4.3131
290	1	0	-1.2575	9.6125	2.9354
291	6	0	1.2226	6.4886	6.681
292	1	0	0.1865	6.186	6.9179
293	1	0	1.7532	6.6259	7.6443
294	1	0	1.7034	5.6468	6.15
295	6	0	-2.6179	7.8511	4.5555
296	1	0	-2.5827	6.788	4.8593
297	1	0	-2.9147	7.879	3.493
298	1	0	-3.4321	8.3457	5.1234
299	6	0	2.6234	8.141	5.5048
300	1	0	3.2521	7.2804	5.2237
301	1	0	3.1328	8.6493	6.3493
302	1	0	2.6091	8.8426	4.6456
303	46	0	0.2263	7.2692	4.0733
304	7	0	1.2224	7.7105	5.8153
305	7	0	-1.2746	8.4804	4.7602
306	6	0	-2.6577	-0.3577	9.1817
307	1	0	-3.2499	-0.0714	8.2922
308	1	0	-1.6344	0.0276	9.0327
309	1	0	-3.0728	0.1786	10.0593
310	6	0	-1.5898	-2.2861	10.2861
311	1	0	-1.6182	-3.3843	10.4275
312	1	0	-1.6698	-1.8298	11.2934
313	1	0	-0.5913	-2.0446	9.8788
314	6	0	-5.0007	-3.748	7.1438
315	1	0	-4.7047	-3.8529	6.084
316	1	0	-5.339	-2.7069	7.2957
317	1	0	-5.8759	-4.4058	7.316
318	6	0	-4.0201	-2.3123	9.7951
319	1	0	-4.1832	-2.1406	10.8929
320	1	0	-4.8145	-1.711	9.2915
321	6	0	-3.3871	-5.4699	7.8579

322	1	0	-3.3344	-5.7416	6.7908
323	1	0	-4.0496	-6.2133	8.3469
324	1	0	-2.3703	-5.5886	8.2855
325	6	0	-4.1822	-3.8025	9.4836
326	1	0	-5.2207	-4.1343	9.746
327	1	0	-3.5223	-4.4179	10.1464
328	46	0	-2.3069	-2.7728	7.5446
329	7	0	-2.6585	-1.8475	9.3329
330	7	0	-3.8357	-4.0516	8.0345
331	6	0	3.7705	-7.24	5.8852
332	1	0	4.3324	-6.3507	5.557
333	1	0	4.5177	-7.9932	6.2092
334	1	0	3.1745	-6.9499	6.7748
335	6	0	-1.0219	-8.0737	3.7127
336	1	0	-0.5986	-7.7335	2.7391
337	1	0	-1.8097	-7.3529	3.9859
338	1	0	-1.5241	-9.045	3.54
339	6	0	2.3071	-9.0869	5.1762
340	1	0	3.0113	-9.9204	4.9195
341	1	0	2.1842	-9.1624	6.2862
342	6	0	-0.5222	-8.2155	6.1236
343	1	0	0.2819	-8.2708	6.883
344	1	0	-1.1748	-9.0982	6.2785
345	1	0	-1.1144	-7.3115	6.3479
346	6	0	3.5556	-7.7738	3.4881
347	1	0	3.9278	-6.7699	3.2167
348	1	0	2.8566	-8.0979	2.6944
349	1	0	4.4206	-8.4663	3.4707
350	6	0	0.9677	-9.3012	4.4672
351	1	0	0.5217	-10.2773	4.7946
352	1	0	1.1123	-9.4082	3.3643
353	46	0	1.2373	-6.4593	4.6648
354	7	0	2.8501	-7.7261	4.8077
355	7	0	0.0586	-8.1268	4.7455
356	6	0	5.4083	-3.8479	7.6383
357	1	0	5.3694	-4.2125	6.599
358	1	0	4.5254	-4.2514	8.174
359	1	0	6.3148	-4.2945	8.0978
360	46	0	3.4925	-1.6512	7.3138
361	7	0	3.7214	-0.4774	8.9807
362	7	0	5.3821	-2.3508	7.6899
363	6	0	2.916	-1.1067	10.0756
364	1	0	3.2653	-2.1403	10.2688
365	1	0	1.8556	-1.1794	9.7752
366	1	0	2.9631	-0.5637	11.0408

367	6	0	6.2958	-1.7683	6.6562
368	1	0	6.3077	-0.666	6.7265
369	1	0	5.9376	-2.0292	5.6414
370	1	0	7.3453	-2.1141	6.7416
371	6	0	5.7623	-1.8855	9.076
372	1	0	5.3752	-2.6068	9.8397
373	1	0	6.8718	-1.8777	9.2407
374	6	0	3.2647	0.9262	8.7286
375	1	0	3.7133	1.3031	7.7916
376	1	0	3.5269	1.6301	9.544
377	1	0	2.1674	0.9669	8.6138
378	6	0	5.1936	-0.4848	9.3202
379	1	0	5.7288	0.2739	8.7011
380	1	0	5.393	-0.1822	10.3826
381	7	0	4.7764	-4.4938	-4.0849
382	7	0	3.6467	-2.7441	4.3625
383	6	0	3.4309	-4.0513	2.2005
384	7	0	2.9954	-4.9712	-2.8894
385	7	0	3.2337	-2.932	5.6235
386	7	0	3.5149	-4.9445	-4.1256
387	7	0	2.4911	-4.6793	4.5232
388	7	0	5.1134	-4.1896	-2.8258
389	6	0	3.1913	-3.8427	3.631
390	6	0	3.9927	-4.4983	-2.0338
391	6	0	4.3898	-3.2747	1.5189
392	1	0	4.9836	-2.5304	2.0631
393	6	0	3.8482	-4.372	-0.5809
394	7	0	2.5566	-4.0856	5.7196
395	6	0	4.5963	-3.4296	0.1452
396	1	0	5.343	-2.8013	-0.3677
397	6	0	2.9229	-5.1801	0.1087
398	1	0	2.3561	-5.9435	-0.4346
399	6	0	2.7044	-5.0157	1.4786
400	1	0	1.9476	-5.6339	1.9862
401	7	0	-0.6447	-1.4955	7.0825
402	7	0	-0.6408	-0.3549	6.3774
403	7	0	3.5187	5.9589	1.9267
404	7	0	2.5623	6.7174	2.478
405	7	0	0.6083	-1.8307	7.421
406	7	0	1.8394	5.9921	3.3403
407	6	0	1.156	1.2366	5.5589
408	6	0	1.9813	3.5336	4.1195
409	6	0	0.2846	2.3311	5.3837
410	1	0	-0.7279	2.2825	5.7924
411	7	0	3.4613	4.7135	2.4243
412	7	0	1.4719	-0.935	6.9282

413	6	0	0.6951	0.032	6.2587
414	6	0	0.6908	3.4659	4.6769
415	1	0	-0.0037	4.3022	4.536
416	6	0	2.4009	4.6988	3.3322
417	6	0	2.4545	1.3149	5.025
418	1	0	3.1437	0.4714	5.1535
419	6	0	2.8583	2.4475	4.3109
420	1	0	3.8626	2.4834	3.8815
421	6	0	1.1758	-3.067	-1.787
422	6	0	-0.1242	-3.0396	-1.3091
423	6	0	-0.9118	-1.8412	-1.4668
424	6	0	-0.3197	-0.7102	-2.0903
425	6	0	1.0274	-0.7803	-2.5639
426	6	0	1.7549	-1.9353	-2.4166
427	1	0	-2.7308	-2.6133	-0.5444
428	1	0	1.7858	-3.9704	-1.6881
429	6	0	-2.2652	-1.742	-1.0116
430	6	0	-1.0789	0.4951	-2.2278
431	1	0	1.4639	0.0974	-3.0433
432	1	0	2.7801	-2.0031	-2.7807
433	6	0	-2.3664	0.5623	-1.7613
434	6	0	-2.9719	-0.575	-1.1539
435	1	0	-0.6091	1.3583	-2.6987
436	1	0	-2.9412	1.483	-1.8439
437	1	0	-4.0039	-0.5004	-0.8058
438	6	0	-0.696	-4.1962	-0.6201
439	1	0	-1.7674	-4.1282	-0.3559
440	6	0	0.0138	-5.2867	-0.274
441	1	0	1.0784	-5.4256	-0.507
442	8	0	0.2256	-7.0454	1.1687
443	8	0	-1.7638	-6.5775	0.4593
444	7	0	-0.5594	-6.3783	0.5043
445	6	0	2.1305	2.3838	0.5182
446	6	0	2.3392	1.0962	0.9775
447	6	0	1.2998	0.4205	1.7117
448	6	0	0.0891	1.1148	1.9744
449	6	0	-0.0903	2.4453	1.4803
450	6	0	0.9042	3.0582	0.7597
451	1	0	2.3764	-1.4568	2.0051
452	1	0	2.9076	2.9111	-0.0383
453	6	0	1.4509	-0.9141	2.2055
454	6	0	-0.9347	0.4737	2.742
455	1	0	-1.0296	2.9625	1.6867
456	1	0	0.7758	4.0686	0.3727
457	6	0	-0.7525	-0.8005	3.2146
458	6	0	0.4507	-1.5086	2.9322

459	1	0	-1.8546	1.0198	2.9467
460	1	0	-1.5126	-1.2834	3.8275
461	1	0	0.5619	-2.5273	3.3062
462	6	0	3.6064	0.4061	0.7257
463	1	0	3.5492	-0.6898	0.6038
464	6	0	4.7907	1.0391	0.6473
465	1	0	4.9212	2.1205	0.7672
466	8	0	6.9608	0.9721	-0.0842
467	8	0	6.0816	-0.884	0.5889
468	7	0	6.0294	0.3187	0.363

References:

1) H. Lee, S. Kang, J. Y. Lee and J. H. Jung, *Soft Matter,* 2012, **8**, 2950.
2) *SMART/SAINT*; *Bruker AXS*, Inc.: Madison, WI, 2004**.**
3) G. M. Sheldrick, *Acta Cryst.* 2008, **A64**, 112-122.
4) G. M. Sheldrick, *SADABS*, University of Göttingen, Göttingen, Germany, 1999.
5) G. M. SHELXL-2013 Sheldrick, University of Göttingen: Göttingen, Germany, 2014.
6) L. J. J. Farrugia, *Appl. Cryst.* 2012, **45**, 849. L. J. Farrugia, *WinGX*, version 2013**.**3; Department of Chemistry, University of Glasgow: Glasgow, Scotland, 2013.
7) P. V.D. Sluis and A. L. Spek, *Acta Cryst*. 1990, **A46**, 194-201.
